# IRF7 deficiency in myeloid cells results in increased disease severity and death during alphavirus encephalomyelitis despite intact expression of type I interferons

**DOI:** 10.1186/s12974-026-03936-7

**Published:** 2026-06-25

**Authors:** Benjamin H. Nguyen, Elise Stanley, Wanmin Dai, Elizabeth M. Troisi, Diane E. Griffin

**Affiliations:** https://ror.org/00za53h95 grid.21107.35 0000 0001 2171 9311 W. Harry Feinstone Department of Molecular Microbiology and Immunology, Johns Hopkins Bloomberg School of Public Health, 615 N Wolfe St, MD 21205 Baltimore, USA

**Keywords:** Sindbis, Interferon, IRF7, Monocyte, CNS

## Abstract

Alphaviruses are arthropod-borne pathogens that cause immune-mediated encephalomyelitis. The prototypic alphavirus, Sindbis virus (SINV), primarily infects neurons in mice, resulting in neurologic disease involving both the brain and spinal cord. Interferon (IFN) regulatory factor (IRF) 7-deficient mice (*Irf7*^−/−^) rapidly succumb to SINV infection with extensive immunopathology while wildtype mice experience only mild disease and recover. IRF7 is widely recognized to be the main transcription factor required for the amplification of type I IFN, specifically IFNα, as *Irf7*^−/−^ mice do not produce IFNα but do express IFNβ. However, based on the current experimental evidence, it is not clear if the severe outcome of SINV infection in *Irf7*^−/−^ mice is due to the absence of IFNα or heretofore unappreciated noncanonical actions of IRF7. In this study, we demonstrate that the administration of IFNα to *Irf7*^−/−^ mice early in infection restricted SINV replication and largely rescued the animals from severe disease and mortality but did not alter the nature of CNS inflammation. Further, Cre-Lox-mediated deletion of *Irf7* in myeloid cells (*Irf7*^fl/fl^Lyz2-cre mice) resulted in severe disease and lethality despite significantly higher peak levels of IFNα than WT animals, suggesting that the lethal phenotype of *Irf7*^−/−^ mice was attributed to the myeloid cell-specific loss of IRF7 and not to the loss of IFNα production. Both *Irf7*^−/−^ and *Irf7*^fl/fl^Lyz2-cre mice had increased levels of monocytes infiltrating into the brain compared to WT, indicating a potential role for IRF7 in the trafficking and activation of myeloid cells. Therefore, while IFNα supplementation may contribute to reducing disease severity by restricting virus replication, disease was ultimately exacerbated by the increased infiltration of inflammatory myeloid cells in *Irf7*^−/−^ mice.

**Supplementary Information:**

The online version contains supplementary material available at https://doi.org/10.1186/s12974-026-03936-7.

## Introduction

Arthropod-borne viruses (arboviruses) are of considerable public health concern due to the expanding range of their vectors [1–4]. Alphaviruses are a group of arboviruses that cause epidemic febrile, arthritic, and encephalitic diseases [5]. The New World alphaviruses are especially dangerous as infection causes encephalomyelitis, which can lead to death or long-term debilitating sequela. Encephalomyelitis can be studied in a mouse model of infection with Sindbis virus (SINV), which is an Old World alphavirus that infects neurons and results in disease similar to its New World counterparts [5–8]. This model of neurotropic SINV infection is useful for understanding immune determinants that drive disease severity and recovery, and insights into these processes are important for the development of treatments or vaccines, which currently do not exist for encephalitic alphavirus infections.

The type I interferons (IFN) make up a broad, innate antiviral defense mechanism that is required for survival from many viral infections [9–11]. The type I IFN family consists mainly of IFNα, which has 13 subtypes in mice, and IFNβ. The family also includes IFN-ε, -κ, -τ, -δ, -ζ, and -ω, but their roles in antiviral mechanisms are not as well defined [12, 13]. Type I IFN is rapidly induced following viral infection and pattern recognition receptor (PRR) engagement. Through their various signaling pathways, PRR activation converges on the phosphorylation of IFN regulatory factor (IRF) 3, which is constitutively expressed in cells. IRF3 activation promotes the expression of IFNβ, which is secreted and signals through the heterodimeric type I IFN receptor, IFNAR1/2. This signaling results in the induction of hundreds of IFN-stimulated genes (ISG) through a JAK/STAT pathway [12–14] that generally have anti-viral and anti-pathogenic effects. An important ISG that is induced is IRF7, which is the master regulator of IFNα expression [15]. For most cells, it is only after IRF7 is induced through IFNβ signaling that IFNα is expressed [14, 15]. The notable exception to this is in plasmacytoid dendritic cells (pDC), which constitutively express IRF7 and are considered the professional IFNα-expressing cell [12, 13]. Interestingly, while IFNα and IFNβ signal through the same receptor, distinct functions for IFNα and IFNβ have been described [16–20]. For example, during chikungunya virus infection, a related alphavirus, IFNα has been reported to be important for restricting virus replication and spread and mitigating inflammation, while IFNβ regulates neutrophil recruitment and promotes inflammation [18, 19]. In the context of SINV infection, while type I IFN is critically important for survival as IFNAR knockout mice rapidly die from infection [11], the specific contributions and nuances of IFNα and IFNβ to this response have not been studied.

The role of IFNβ can be studied using *Ifnb*^−/−^ mice [21] because IFNβ is encoded by a single gene; however, there are multiple genes encoding the multiple subtypes of IFNα, so full knockout mice cannot be easily generated. Therefore, *Irf7*^−/−^ mice have been used as a surrogate for IFNα-deficiency [15] because *Irf7*^−/−^ mice do not express IFNα but have intact IFNβ production [11, 15]. However, the interpretation of findings of *Irf7*^−/−^ experiments depend on additional validation, for example, by antibody-mediated IFNα-depleting procedures. This is not possible in all contexts, including in studies of the central nervous system (CNS), because antibodies do not easily cross the blood-brain barrier (BBB) [22]. As such, it becomes difficult to conclude whether CNS-specific observations in *Irf7*^−/−^ mice are due to the loss of IFNα or the loss of IRF7. This is particularly important because, despite their name, the IRFs have other roles aside from regulating IFN induction [23]. The most well-documented, non-canonical role for IRFs is the regulation of myeloid cell development and polarization. In the brain, IRF7 regulates the polarization of both resident and infiltrating myeloid cells, but the results are confounding. Knock-down of *Irf7* in microglial cells promotes pro-inflammatory polarization in some contexts but reparative polarization in others [24, 25]. However, other studies examining IRF7-NF-κB interactions demonstrate that IRF7 is needed for suppression of NF-κB-induced inflammation [26–28], suggesting that IRF7 plays a regulatory role in controlling pro-inflammatory polarization, possibly to prevent excessive immune-mediated damage in response to viral infections. Due to the various functions of IRF7 that are independent of inducing IFNα, it is important to properly examine whether the phenotypes seen with *Irf7*^−/−^ mice are due to IRF7 or IFNα deficiency.

To tease apart the individual contributions of IRF7 and IFNα in the pathogenesis of SINV-induced encephalomyelitis, we expanded upon our previous studies showing that following infection with the TE strain of SINV (SINV TE), *Irf7*^−/−^ mice experienced a rapid onset of neurologic disease with severe bilateral hindlimb paralysis and death while wildtype (WT) animals had only mild disease and fully recovered [11, 29]. Death in *Irf7*^−/−^ mice was associated with exacerbated CNS inflammation that is characterized by infiltrating inflammatory myeloid cells [11, 29]. Here, we report that early treatment of *Irf7*^−/−^ mice with exogenous IFNα suppressed virus replication but did not completely rescue the death phenotype nor dampen the levels of inflammatory cytokine production or cellular infiltration. These results suggest additional roles for IRF7 during SINV TE infection. In approaches employing cell type-specific deletion of *Irf7* in astrocytes, neurons, or myeloid cells, only genetic deletion of *Irf7* in myeloid cells (*Irf7*^fl/fl^*Lyz2*-cre) resulted in elevated viral titers, disease severity, and lethality that resembled full-body *Irf7*^−/−^ mice. Importantly, unlike *Irf7*^−/−^ animals, the *Irf7*^fl/fl^*Lyz2*-cre mice expressed levels of IFNα that exceeded the WT controls, suggesting that the disease phenotype seen in *Irf7*^−/−^ mice is largely driven by the loss of IRF7 in myeloid cells and not the loss of IFNα production. The *Irf7*^fl/fl^*Lyz2*-cre mice had comparable monocyte infiltration into the CNS to that of *Irf7*^−/−^ mice. These results strongly indicate a noncanonical role for IRF7 in myeloid cells that is critical for controlling virus replication and lethal pathology in the CNS and point to IRF7 as a novel target for drug development.

## Methods

### Mice, virus infections, and treatments

Wildtype (WT) C57BL/6J, *Syn1*-cre, and *Gfap*-cre mice were obtained from Jackson Laboratories. *Irf7*^-/-^ mice were a gift from Michael Diamond (Washington University, St. Louis, MO, USA) and *Lyz2*-cre (*LysM*-cre) mice were a gift from Daniela Cihakova (Johns Hopkins University, Baltimore, MD, USA). All mice were bred in house and transgenic mice were on a C57BL/6J background.

SINV TE [30] was grown on BHK-21 cells and quantified by plaque formation. For infection, 5-8-week-old mice of both sexes were deeply anesthetized with isoflurane and intracranially inoculated in the left cerebral hemisphere with 10^3^ plaque-forming units (PFU) of SINV TE in 20 µL of phosphate-buffered saline (PBS).

For clinical evaluation, mice were weighed daily and scored for signs of neurologic disease using the following 6-point system: 0 = clinically normal; 1 = abnormal gait and/or ataxia; 2 = mild hunched posture with occasional two-leg stance but normal demeanor and ambulation; 3 = markedly hunched posture with minimal ambulation or obtunded; 4 = unilateral hindlimb paralysis; 5 = bilateral hindlimb paralysis or moribund; 6 = death.

Mice treated with IFNα were given 10^5^ IU of recombinant mouse IFNα (Miltenyi Biotec) in 100 µL PBS via intraperitoneal injection for days 1–10 post-infection. All experiments were performed in accordance with protocols approved by the institutional animal care and use committee of Johns Hopkins University.

For tissue collection, mice were euthanized by isoflurane inhalation and perfused with PBS. Brains and spinal cords were collected, flash-frozen, and stored at -80 °C.

### Virus quantification from CNS tissues

Frozen tissues were homogenized at 20% weight/volume (w/v) in PBS using Lysing Matrix A tubes (MP Biomedicals) for 40 s at 6.0 M/s in a FastPrep-24 homogenizer (MP Biomedicals). Homogenates were serially diluted in Dulbecco’s modified Eagle’s medium (Gibco) + 1% heat-inactivated fetal bovine serum (FBS; Gibco). Samples were incubated on BHK-21 cells for 1 h at 37 °C with occasional shaking. Cells were overlaid with agarose containing 2X minimal essential media (Gibco) + 2% FBS, incubated at 37 °C, 5% CO_2_ for 48 h, and stained with 10% neutral red (Sigma Aldrich). Plaques were counted and presented as log_10_PFU per gram of tissue.

### RNA sequencing

Brains from age-matched WT and *Irf7*^-/-^ mice (two male, one female) were collected and flash frozen in QIAzol (Qiagen). For total RNA isolation, frozen tissues were homogenized in QIAzol using Lysing Matrix D tubes (MP Biomedicals) for 40 s at 6.0 M/s in a FastPrep-24 homogenizer (MP Biomedicals). RNA was extracted with chloroform, precipitated with isopropanol, cleaned up with the RNeasy Plus Mini kit (Qiagen). RNA was submitted for library preparation, quality control (QC), and sequencing to Admera Health (South Plainfield, NJ). RNA sample quality was assessed by High Sensitivity RNA Tapestation (Agilent Technologies) and quantified by AccuBlue Broad Range RNA Quantitation assay (Biotium). Libraries were constructed with KAPA RNA HyperPrep with RiboErase (Roche) and performed based on manufacturer’s recommendations. Final library quantity was measured by KAPA SYBR FAST qPCR and library quality evaluated by TapeStation D1000 ScreenTape (Agilent Technologies). Final library size was approximately 430 base pairs (bp) with an insert size of approximately 200 bp, and 8 nucleotide dual indices (Illumina) were used. Equimolar pooling of libraries was performed based on QC values and sequenced on an Illumina NovaSeq X Plus (Illumina) with a read length configuration of 150 bp for 60 million paired end (PE) reads per sample (30 million in each direction).

QC reports for raw sequencing reads were generated with FastQC and showed no irregularities. Reads were then trimmed with fastp and aligned to the reference genome using STAR. Gene counts were quantified using Kallisto and differential gene expression analysis was done using DESeq2. Downstream analyses were done with the following R packages: goseq, enrichKEGG, and ReactomePA. Genes were considered differentially expressed if they were > 1.5- or <-1.5-fold change and reached a significance cut-off of p-value = 0.01.

### Real-time quantitative Reverse Transcription PCR (qRT-PCR)

For total RNA isolation, frozen tissues were homogenized in QIAzol (Qiagen) using Lysing Matrix D tubes (MP Biomedicals) for 40 s at 6.0 M/s in a FastPrep-24 homogenizer (MP Biomedicals). RNA was extracted with chloroform, precipitated with isopropanol, cleaned up with the RNeasy Plus Mini kit (Qiagen), and quantified with a nanodrop spectrophotometer. cDNA was synthesized using the SuperScript III First-Strand Synthesis System (Invitrogen) with normalized RNA concentrations across samples and oligo dT primers according to the manufacturer’s instructions. The cDNA was diluted, and qRT-PCR was performed using PrimeTime gene expression assays (Integrated DNA Technologies) and EagleTaq Universal Master Mix (Roche). *Gapdh* mRNA levels were determined using the TaqMan Rodent GAPDH Control Reagents (Applied Biosystems). The target gene Ct value was normalized to the Ct value of *Gapdh*. This normalized value was used to calculate the gene expression level relative to the average of the uninfected control value.

SINV RNA quantification was performed using the following primers and probes specific for SINV *nsP2*: primer nsP2 3373 F (5′-CCG CAA GTA TGG GTA CGA TCA-3′), primer nsP2 3454R (5′-GTG CCC TTC CCA GCT AGC T-3′), and TaqMan probe nsP2 3317 (5′–6-carboxyfluorescein [6-FAM]–CCA TTG CCG CCG AAC TCT CCC–6-carboxytetramethylrhodamine [6-TAMRA]–3′). RNA copy numbers were quantified using a standard curve consisting of 10-fold serial dilutions ranging from 3 × 10^7^ to 300 copies of the pCRII-TOPO plasmid containing the SINV nsP2 region and normalized to those for *Gapdh*. All reactions were run on an Applied Biosystems 7500 real-time PCR machine.

### Enzyme-Linked Immunosorbent Assay (ELISA)

Protein homogenates were generated from frozen tissues as described above with the addition of cOmplete Protease Inhibitor Cocktail (Roche) and PhosSTOP phosphatase inhibitor (Roche). Commercial ELISA kits were used to measure IFNα (PBL Assay Sciences), IFNβ (PBL Assay Sciences), and IFNγ (BioLegend) levels according to manufacturer recommendations. Samples were originally tested at 1:2 dilution in duplicate and re-run with further dilution if protein levels exceeded the upper limit of detection of the assay.

Anti-SINV antibodies were measured by ELISA using Nunc Maxisorp 96-well plates (Thermo Scientific) coated with 10^6^ PFU of SINV in coating buffer (50 mM NaHCO_3_ pH 9.6) overnight at 4 °C and subsequently blocked with 10% FBS in PBS + 0.05% Tween-20 (PBS-T) for 2 h at 37 °C. Brain homogenates were diluted 1:3 in PBS-T + 10% FBS and incubated overnight at 4 °C. Plates were incubated with HRP-conjugated goat anti-mouse IgM or IgG (Southern Biotech) diluted 1:1000 in PBS-T + 10% FBS for 2 h at room temperature (RT) and developed using BD OptEIA TMB Substrate Reagent kit with 2M H_2_SO_4_ as stop solution. Plates were read at 450 nm, and optical density (OD) values for blank wells were subtracted from OD values of each sample.

### Mononuclear cell isolation

Single-cell suspensions were made from freshly isolated brains by homogenizing three times in RPMI containing 10% FBS, 1 mg/mL collagenase IV (Worthington Biochemical Corporation), and 0.1 mg/mL DNase I (Stem Cell Technologies) using the GentleMACS system (Miltenyi), employing 15 min incubations at 37 °C with gentle agitation between each round of homogenization. Homogenates were filtered through a 70 μm filter and washed with RPMI containing 10% FBS. Myelin debris and erythrocytes were removed by centrifugation on a 30/70% Percoll gradient for 30 min at 4 °C. Mononuclear cells were collected at the interface, residual erythrocytes lysed with ACK lysing buffer (Quality Biological), and cells washed several times with RPMI containing 10% FBS before freezing in a 10% DMSO and 90% FBS solution.

### Flow cytometry

Cells were thawed, washed, and counted, and 10^6^ cells were plated to be stained with the LIVE/DEAD Fixable Near-IR 633 dead cell stain (Invitrogen) in PE buffer (1X PBS, 2 mM EDTA), blocked with rat anti-mouse CD16/CD32 (BD Pharmingen, clone 2.4G2) diluted in PE buffer, and surface stained with an antibody cocktail in FACS buffer (1X PBS, 2 mM EDTA, 0.5% BSA) for 30 min at RT. The following antibodies from BioLegend or BD Pharmingen were used for surface staining: CD45 (clone 30-F11), CD11b (clone M1/70), F4/80 (clone QA17A29), CD86 (clone GL-1), I-A/I-E (MHC-II; clone M5/114.15.2), CD206 (clone C068C2), Ly6C (clone HK1.4), CD43 (clone S7), CD11c (clone N418), CD103 (clone 2E7), SiglecH (clone 551), CD3 (clone 17A2), CD4 (clone GK1.5), CD183 (CXCR3; clone S18001A), CD8a (clone 53 − 6.7), NK1.1 (clone S17016D), CD19 (clone 6D5), CD23 (clone B3B4), IgM (clone RMM-1), CD5 (clone 53 − 7.3), and Ly6G (clone 1A8).

Following staining, cells were washed, suspended in FACS buffer, and data were acquired on a BD FACSymphony A5-SE spectral flow cytometer (Johns Hopkins Bloomberg Flow Cytometry and Immunology Core) with FACS Diva software (version 9.0) and analyzed using FlowJo (version 10.10). Aberrant events were removed by the FlowAI plugin, and gating was based on fluorescence minus one (FMO). From live CD45^+^ cells, the populations were defined as outlined in Table S1.

### Generation of *Irf7*^fl/fl^ mice

*Irf7* ^fl/fl^ mice were created as described [31, 32]. Briefly, CRISPR RNAs (crRNA) were designed to target the introns between exons 1 and 2 (5’-TAAGGGTCGGGTGTAGTTTG), and 4 and 5 (5’-GCCCAAGGCTCTGACTTAGC), so that cre-mediated disruption of the *Irf7* gene would be similar to original *Irf7*^-/-^ mice [15] (Figure S2A). A single-stranded DNA (ssDNA) molecule (Figure S2D) was designed by inserting LoxP sites within crRNA-targeting regions of the loci to prevent subsequent cuts by the Cas9 enzyme. Both crRNAs and the ssDNA molecule were synthesized by Integrated DNA Technologies (Coralville, Iowa) and the Cas9 enzyme and tracrRNA were provided by the Johns Hopkins Transgenic Core Laboratory. All CRISPR reagents were microinjected into C57BL/6J mouse zygotes, and embryos were transferred into pseudopregnant mice by the Transgenic Core. Resulting offspring were genotyped to identify transgenic founders, backcrossed to C57BL/6J for multiple generations, and bred in-house as homozygotes. Genotyping for the 5’ LoxP site was done with the following primers: 5’-GGGAAAUUCUUGUCCCCUCUAC and 5’-CUAAACAGUCUAAACAGCGCCC, and genotyping for the 3’ LoxP site was done with the following primers: 5’-GGTGGTTTCAATGGTTTGATTT and 5’-CACTTCTTCCCTATTTTCCGTG (Figure S2A). Successful Cre-mediated recombination resulted in excision of exons 2–4, thus mimicking the genetics of the full-body *Irf7*^-/-^ mice [15]. Cre recombination was confirmed by PCR (Figure S2B) using the aforementioned primers along with 5’-GTGGTACCCAGTCCTGCCCTCTTTATAATCT and 5’-AGTAGATCCAAGCTCCCGGCTAAGTTCGTAC (Figure S2C) that flanked two regions outside of LoxP sites. PCR used the GoTaq Green master mix (Promega), and amplicons were purified and sequence-verified for LoxP inserts by the Johns Hopkins Synthesis and Sequencing Core.

### Statistics

Statistical analyses were performed using GraphPad Prism 10. The qRT-PCR, viral titer, and ELISA assays were analyzed for significant differences at each timepoint by two-way analysis of variance (ANOVA) with Tukey’s (three genotypes) or Bonferroni’s (two genotypes) multiple comparisons post-test. Flow cytometry assays were analyzed by one-way ANOVA with Tukey’s multiple comparisons test. Survival was assessed using a Kaplan–Meier estimator and the log-rank test with Bonferroni correction for multiple comparisons. A P-value of < 0.05 was considered significant for all tests unless adjusted for survival analyses.

## Results

### IRF7 deficiency results in increased inflammatory gene expression in the brain

In our previous studies, *Irf7*^−/−^ mice develop severe bilateral hindlimb paralysis and death around 7 days post-infection (dpi) with SINV TE, which is in contrast to the mild disease and subsequent recovery observed in WT mice [11]. The *Irf7*^−/−^ phenotype was associated with increased viral replication in motor neuron rich areas of the CNS as well as increased myeloid cell infiltration into the brain and spinal cord [29]. To further understand the breadth of changes to the immune response in the absence of *Irf7*, the brains of uninfected (0 dpi), 3 dpi, and 5 dpi WT and *Irf7*^−/−^ mice were analyzed using bulk RNA sequencing (Fig. 1, Figure S1). These time points were chosen based on the findings that 3 dpi was the peak of type I IFN expression, and 5 dpi was the time of paralysis onset [11]. The number of unique differentially expressed genes (DEG) in *Irf7*^−/−^ mice compared to WT at 0 and 3 dpi were low in number, 11 and 13, respectively. In stark contrast, at 5 dpi the brains of *Irf7*^−/−^ mice had 2,830 unique DEGs compared to the brains of 5 dpi WT mice (Fig. 1A). This finding was underscored by principal component analysis (PCA) (Fig. 1B), which showed 5 dpi *Irf7*^−/−^ mice clustering distinctly from 5 dpi WT mice, suggesting that IRF7 deficiency during SINV TE infection resulted in a distinct transcriptomic profile that could underlie differences in disease severity. Similarly, KEGG pathway and Reactome analyses showed increases to immune-related pathways in *Irf7*^−/−^ mice compared to WT – particularly those involving cytokine signaling – such as cytokine-cytokine receptor interaction, interleukin signaling, and tumor necrosis factor (TNF) signaling (Table 1). Due to the high rank of cytokine signaling in pathway analyses, expression of 227 genes encoding core cytokines were examined, with 59/227 genes showing differential expression over the experimental time course (Fig. 1C). *Irf7*^−/−^ mice had increased expression of inflammatory chemokines and genes from the TNF super family. Taken together, these results show elevated levels of inflammation associated with the increased disease severity seen in *Irf7*^−/−^ mice; however, it remains unclear whether these changes in gene expression are a consequence of IRF7 deficiency or due to the absence of IFNα production and its downstream signaling.

**Fig. 1:**
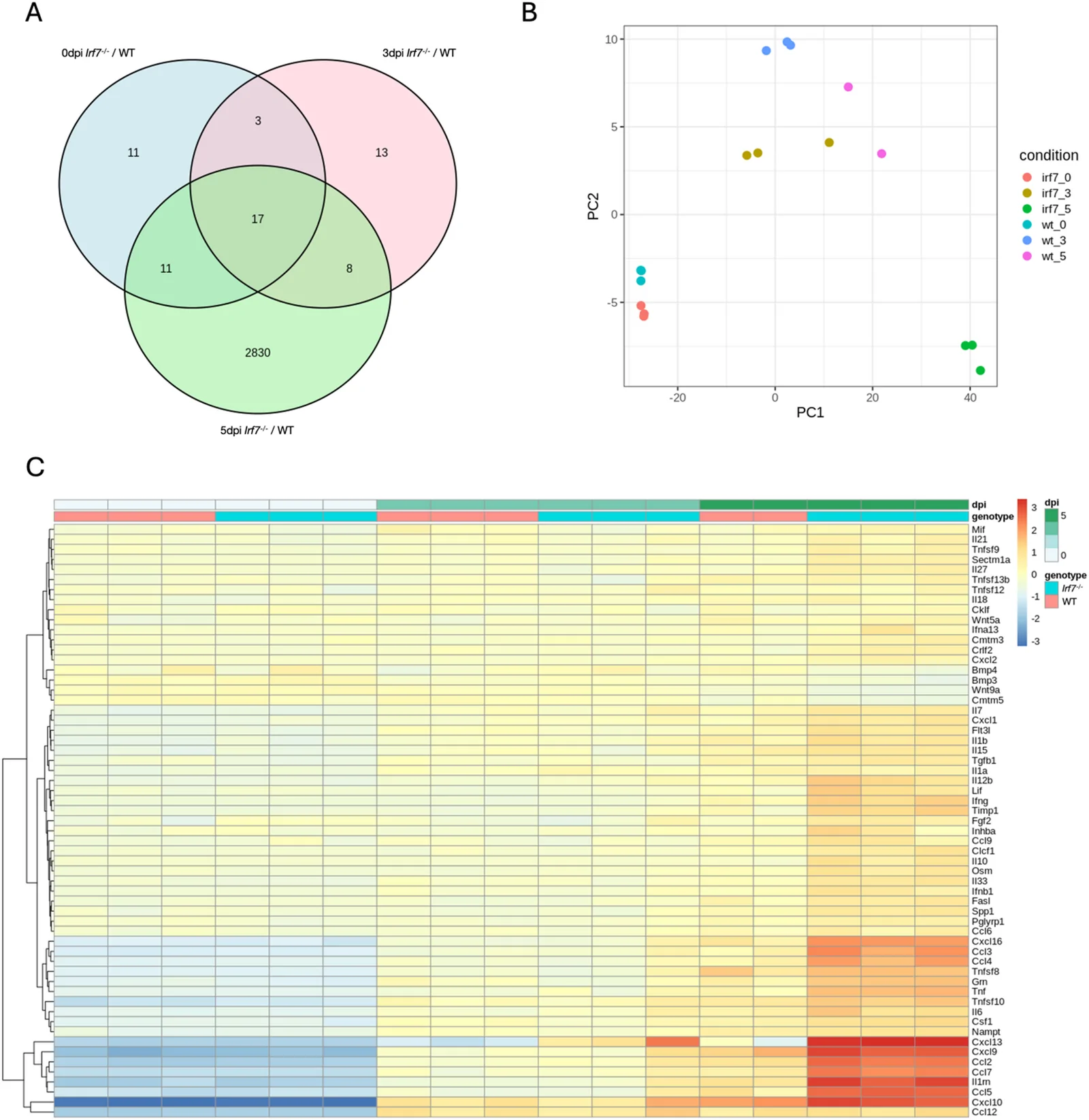
RNA sequencing in the brains of WT and Irf7^−/−^ mice. RNA from the brains of uninfected, 3 dpi, and 5 dpi WT and Irf7^−/−^ mice was extracted for total RNA sequencing. (**A**) Venn diagram showing the number of DEGs in Irf7^−/−^ mice compared to WT mice at 0, 3, and 5 dpi. DEGs and fold changes are comparing Irf7^−/−^ mice to time-matched WT mice. (**B**) Principal component analysis (PCA) comparing each condition (genotype and dpi). (**C**) Heatmap showing statistically significant differentially regulated cytokines in the dataset from a list of 227 common cytokines, striated by individual samples and grouped by genotype and infection status. Fold change is represented as a gradient from blue (down-regulation) to red (up-regulation). Data are from three at mice per time point per genotype

**Table 1:**
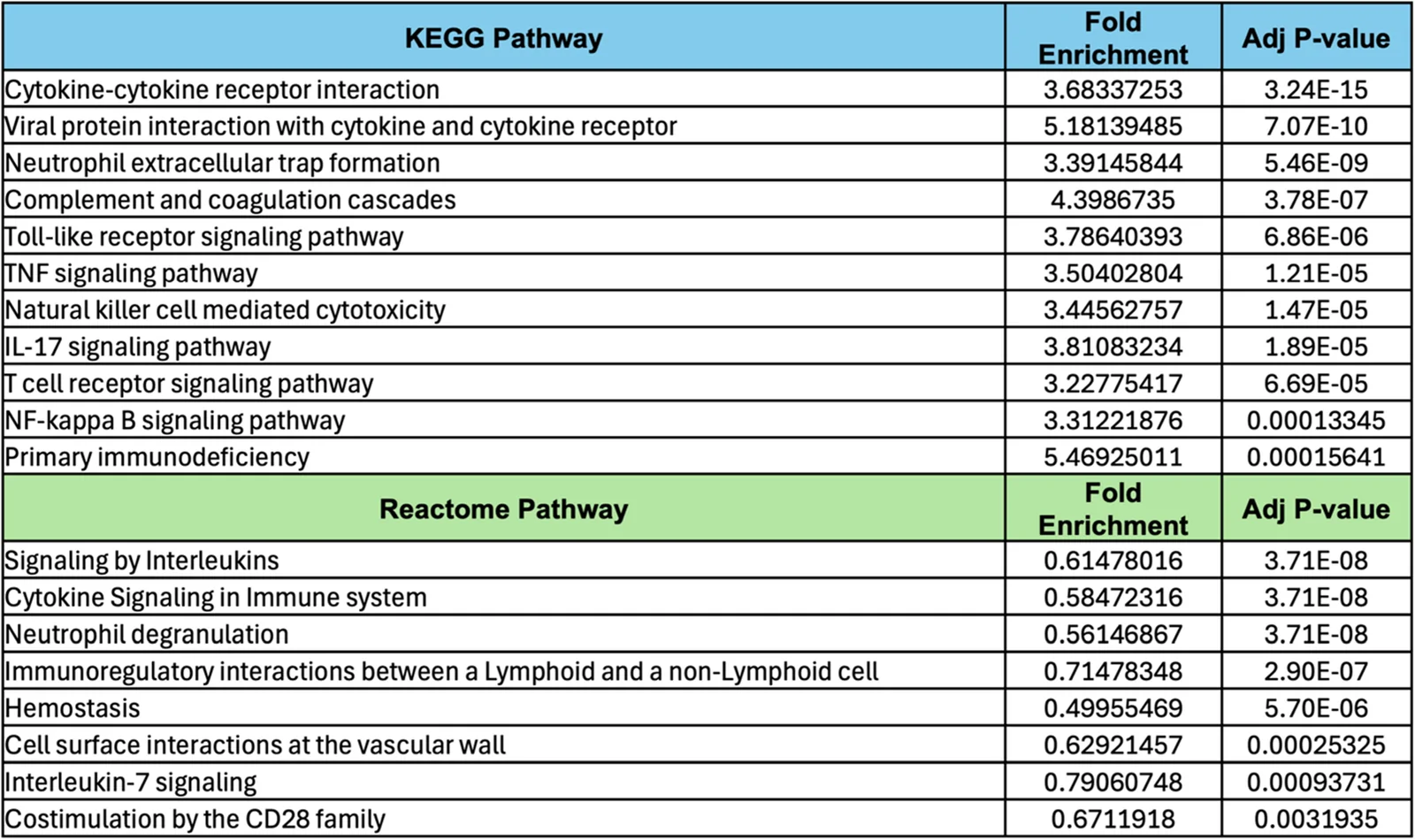
Pathway analyses of differentially expressed genes

The top enriched KEGG and Reactome pathways, fold enrichment, and P-values as represented by DEGs that were upregulated in Irf7−/− mice compared to WT mice at 5dpi

### IFNα restricts virus replication but does not modulate inflammation in *Irf7*^−/−^ mice

IRF7 is considered the master regulator of IFNα [15] and, while *Irf7*^−/−^ mice lack IFNα expression, IFNβ expression is retained [11]. Therefore, to determine the contribution of IFNα during the host response to SINV TE infection and gain an insight into the relative contributions of IRF7 and IFNα to the disease phenotype in *Irf7*^−/−^ mice, we treated *Irf7*^−/−^ mice with exogenous IFNα daily for days 1–10 post SINV TE infection, a regimen which has been shown to efficiently cross the BBB [33] (Fig. 2A and C). Treatment of infected *Irf7*^−/−^ mice with IFNα resulted in less weight loss from baseline, but the weight changes were still greater than the control infected WT mice (Fig. 2A). Similarly, IFNα treatment reduced disease severity and prevented infected *Irf7*^−/−^ mice from developing paralysis, but disease was still more severe than infected WT mice (Fig. 2B). Most strikingly, supplementation with IFNα rescued roughly half of the mice from dying (Fig. 2C), suggesting that the loss of IFNα expression in *Irf7*^−/−^ mice accounts for some protection from the morbidity and mortality observed in the SINV TE-infected WT animals.

**Fig. 2:**
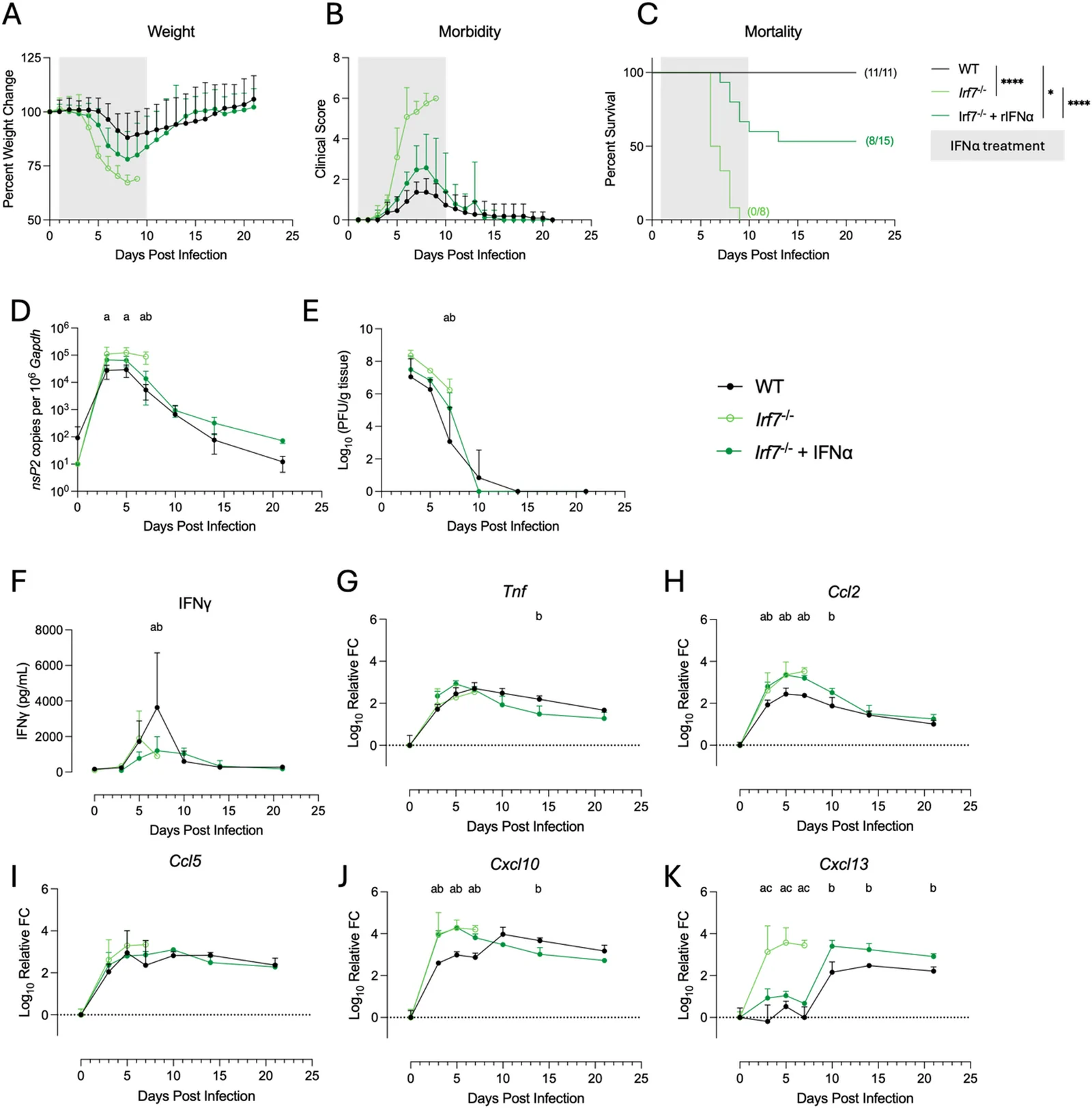
Disease manifestation, viral kinetics, and cytokine response of Irf7^−/−^ mice treated with IFNα. 5-8-week-old male and female WT, Irf7^−/−^, or IFNα-treated Irf7^−/−^ mice were intracerebrally inoculated with 10^3^ PFU of SINV TE. IFNα-treated mice were administered IFNα i.p. at days 1–10 post-infection. Mice were monitored daily for signs of weight loss (**A**), clinical disease (**B**), and death (**C**). The clinical scoring system was: 0 = clinically normal; 1 = abnormal gait and/or ataxia; 2 = mild hunched posture with occasional two-leg stance but normal demeanor and ambulation; 3 = markedly hunched posture with minimal ambulation or obtunded; 4 = unilateral hindlimb paralysis; 5 = bilateral hindlimb paralysis or moribund; 6 = death. Survival was assessed using a Kaplan–Meier analysis and the log-rank test with Bonferroni correction for multiple comparisons (α_Bonferroni_ = 0.016). No indicator, non-significant, *P < 0.016, **P < 0.01, ***P < 0.001, ****P < 0.0001. Virus titers in the brains were assessed by qRT-PCR (**D**) and plaque formation (**E**). Data are presented as the mean ± SD from three or four independent experiments (plaque assay) or three individual mice per time point per genotype (qRT-PCR). Inflammatory cytokine (**F**, **G**) and chemokine (**H**-**K**) expression in the brain was measured by ELISA (**F**) or qRT-PCR (**G**-**K**). qRT-PCR expression was normalized to Gapdh and compared to uninfected controls of each genotype. Data are presented as the mean ± SD for three-four individual mice per time point per genotype. Data labeled with letters differ significantly at that time point (*P* < 0.05). No indicator: non-significant, a: WT vs. Irf7^−/−^, b: WT vs. Irf7^−/−^ + IFNα, c: Irf7^−/−^ vs. Irf7^−/−^ + IFNα, d: three-way difference; Tukey’s (0–7 dpi) or Bonferroni’s (9–21 dpi) multiple comparisons test

Viral RNA levels in the brain were highest in *Irf7*^−/−^ mice and lowered to near WT levels when *Irf7*^−/−^ mice were treated with IFNα (Fig. 2D). Similarly, titers of infectious virus were highest in *Irf7*^−/−^ mice and lowered to near WT levels when treated with IFNα, with surviving mice clearing virus by 10 dpi, similar to WT mice (Fig. 2E). These findings indicate that IFNα is important for efficient restriction of SINV TE replication in the CNS.

Further, because disease from SINV TE infection is immune-mediated [34–36], and *Irf7*^−/−^ mice have heightened inflammation following SINV TE infection [11, 29], we assessed how IFNα treatment influences inflammation in the CNS. To that end, inflammatory cytokines and chemokines were measured (Fig. 2F and K). IFNγ protein levels were measured because it is an important cytokine for resolution of SINV infection [37, 38], and IFNα treatment of *Irf7*^−/−^ mice did not raise IFNγ levels to that seen in infected WT control animals (Fig. 2F). Similarly, the expression levels over time of *Tnf*, *Ccl2*, *Ccl*, and *Cxcl10* were elevated in the brains of the IFNα treated group and not significantly different from the *Irf7*^−/−^ animals (Fig. 2G and J). In contrast, the expression levels of *Cxcl13* were significantly lower with IFNα treatment than that observed in the infected *Irf7*^−/−^ mice (Fig. 2K). Taken together, these data demonstrate that, while important for early control of virus replication, IFNα is not the sole regulator of the inflammatory response to SINV TE infection. Additionally, the data support the idea that IRF7 has direct mechanistic roles in antiviral responses beyond its ability to induce IFNα production.

### Myeloid-specific deletion of *Irf7 *results in mortality from SINV TE infection

To further study the role of IRF7 in death and severe disease, we first sought to determine whether there was a cell type-specific requirement for IRF7. We generated *Irf7* floxed mice (*Irf7*^fl/fl^) by inserting LoxP sites between the first and second, and fourth and fifth, exons of the *Irf7* gene (Figure S2) [31, 32]. The *Irf7*^fl/fl^ mice were crossed with three different Cre driver lines: *Irf7*^fl/fl^*Gfap*-cre for astrocytic-specific knockout, *Irf7*^fl/fl^*Syn1*-Cre for neuronal-specific knockout, and *Irf7*^fl/fl^*Lyz2*-cre for myeloid-specific knockout. Knockout mice at 5–8 weeks of age were infected with SINV TE, and initial screening was based on mortality (Fig. 3A). Despite a small number of deaths, *Irf7*^fl/fl^, *Irf7*^fl/fl^*Gfap*-cre, and *Irf7*^fl/fl^*Syn1*-cre mice generally survived infection through the end of the 21-day observation period and the mean survival times were comparable to WT and not statistically significantly different. In striking contrast, the myeloid-specific knockout of *Irf7* resulted in 78% of mice succumbing to SINV TE infection by 9 dpi, which closely resembled the mortality seen in *Irf7*^−/−^ mice. These findings strongly suggest that IRF7-deficiency in myeloid cells significantly contributes to the phenotype of the global *Irf7* knockout. Due to similarities in the extent and kinetics of mortality, *Irf7*^fl/fl^*Lyz2*-cre mice were monitored for weight change and neurologic disease (Fig. 3B and C). Percent weight change from baseline in *Irf7*^fl/fl^*Lyz2*-cre mice was nearly identical to *Irf7*^−/−^ mice, and *Irf7*^fl/fl^*Lyz2*-cre mice that survived took longer to recover to original starting weights compared to WT mice. When assessing for signs of neurologic disease, *Irf7*^fl/fl^*Lyz2*-cre mice that survived (Fig. 3C; solid line) had mild neurologic disease comparable to the infected WT mice. However, the *Irf7*^fl/fl^*Lyz2*-cre mice that succumbed to infection (Fig. 3C; dashed line) developed paralysis and death, albeit the paralysis phenotype was not as severe as *Irf7*^−/−^ mice. The lack of uniform mortality in *Irf7*^fl/fl^*Lyz2*-cre mice suggests that other factors besides myeloid expression of IRF7 may contribute to death in *Irf7*^−/−^ mice. Nonetheless, these results strongly suggest that the loss of IRF7 expression in myeloid cells is associated with more severe disease outcomes from SINV TE infection.

**Fig. 3:**
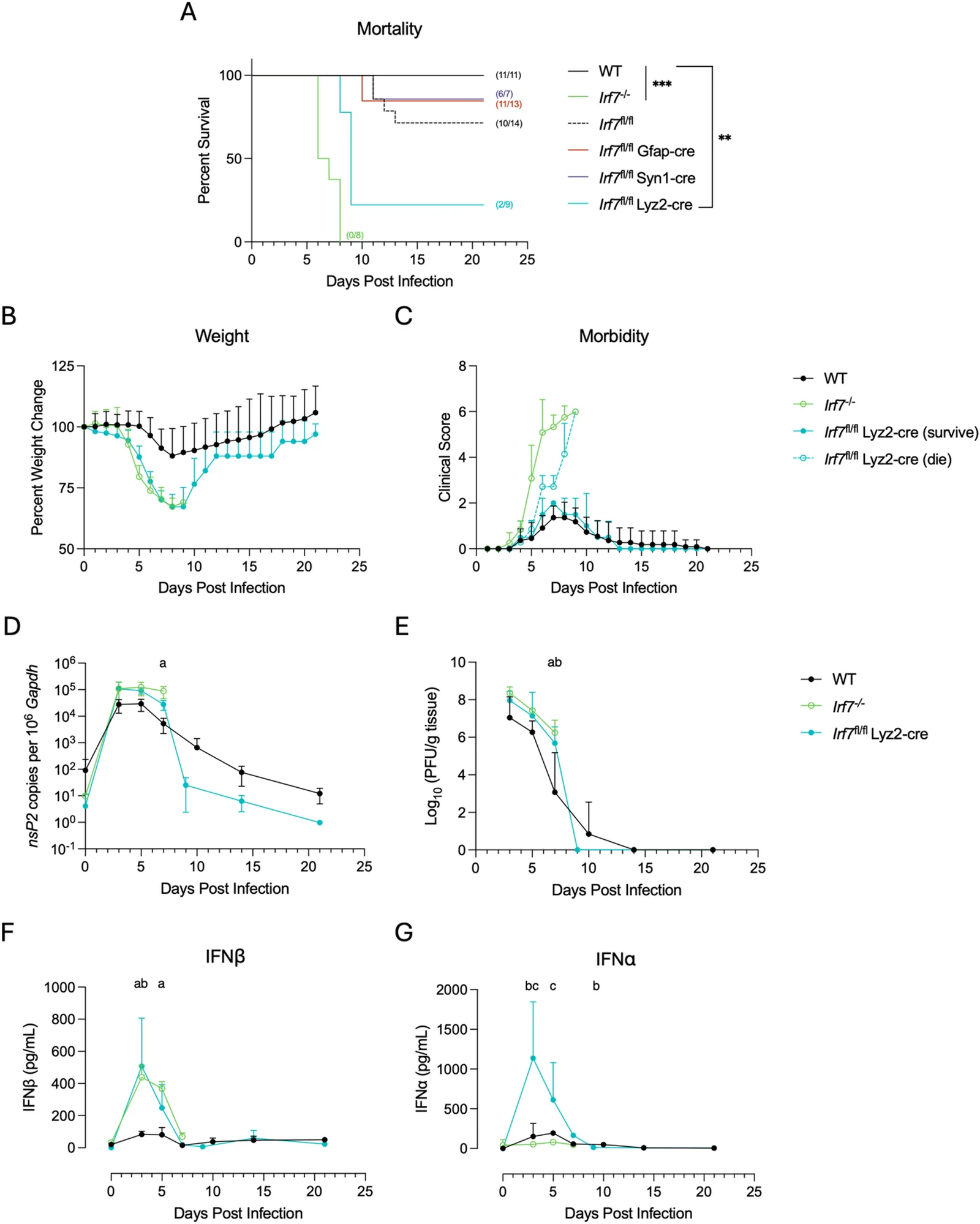
Disease manifestation in astrocyte-, neuronal-, and myeloid-specific knockouts of Irf7. 5-8-week-old male and female WT, Irf7^−/−^, and Irf7^fl/fl^ mice with Gfap-, Syn1-, or Lyz2-cre drivers were intracerebrally inoculated with 10^3^ PFU of SINV TE. Mice were monitored daily for signs of death (**A**), weight loss (**B**), and clinical disease (**C**). For Irf7^fl/fl^ Lyz2-cre mice, disease manifestation for mice that survived infection are represented with the solid line and closed circles, whereas mice that succumbed to infection are represented with the dashed line and open circles (**C**). The clinical scoring system was: 0 = clinically normal; 1 = abnormal gait and/or ataxia; 2 = mild hunched posture with occasional two-leg stance but normal demeanor and ambulation; 3 = markedly hunched posture with minimal ambulation or obtunded; 4 = unilateral hindlimb paralysis; 5 = bilateral hindlimb paralysis or moribund; 6 = death. Survival was assessed using a Kaplan–Meier curve and the log-rank test with Bonferroni correction for multiple comparisons (α_Bonferroni_ = 0.01). No indicator, non-significant, *P < 0.01, **P < 0.001, ***P < 0.0001. Viral replication (**D**, **E**) and type I IFN expression (**F**, **G**) was assessed in the brains of mice after SINV infection. Virus titers in the brains were assessed by qRT-PCR (**D**) and plaque formation (**E**). IFNβ and IFNα levels were measured via ELISA (**F**, **G**). Data are presented as the mean ± SD from three-four mice from three independent experiments (plaque assay) or three-four individual mice per time point per genotype (qRT-PCR and ELISA). Data labeled with letters differ significantly at that time point (*P* < 0.05). No indicator: non-significant, a:WT vs. Irf7^−/−^, b: WT vs. Irf7^fl/fl^ Lyz2-cre, c: Irf7^−/−^ vs. Irf7^fl/fl^ Lyz2-cre, d: three-way difference; Tukey’s (0–7 dpi) or Bonferroni’s (9–21 dpi) multiple comparisons test

### IRF7 knockout in myeloid cells results in altered virus replication and type I IFN expression

SINV TE replication kinetics were measured in the brains of WT, *Irf7*^−/−^, and *Irf7*^fl/fl^*Lyz2*-cre mice. Copies of viral genomic RNA (Fig. 3D) were elevated in both *Irf7*^−/−^ and *Irf7*^fl/fl^*Lyz2*-cre mice compared to WT at 3 and 5 dpi but did not reach statistical significance. However, at 7 dpi, *Irf7*^−/−^ mice had sustained viral RNA replication at the time of death whereas *Irf7*^fl/fl^*Lyz2*-cre mice started to clear viral RNA with kinetics similar to WT mice. Regarding infectious virus (Fig. 3E), *Irf7*^fl/fl^*Lyz2*-cre mice had increased viral titers compared to WT animals, which mirrored titers seen in the *Irf7*^−/−^ group. These titers were significantly elevated at 7 dpi compared to WT mice, but *Irf7*^fl/fl^*Lyz2*-cre mice that survived go on to clear virus from the brain around 9 dpi, which is consistent with clearance dynamics of WT mice. This indicated that myeloid expression of IRF7 is partly responsible for initial restriction of virus replication in the CNS but has no impact on the ability to ultimately clear the virus in survivors.

Additionally, type I IFN levels were measured in the brains of infected animals to determine whether IRF7-deficient myeloid cells affected the overall type I IFN response in the CNS (Fig. 3F and G). While *Irf7*^fl/fl^*Lyz2*-cre mice produce high amounts of IFNβ similar to the levels seen in *Irf7*^−/−^ mice (Fig. 3G), notably, *Irf7*^fl/fl^*Lyz2*-cre mice also produced IFNα at levels that far exceeded the WT and *Irf7*^−/−^ controls (Fig. 3F). This indicates that IRF7-expressing myeloid cells play a heretofore unappreciated role in regulating the overall levels of type I interferon produced in the CNS in the context of SINV TE infection.

In contrast to the heightened IFNα and IFNβ levels associated with ablating *Irf7* expression in myeloid cells, SINV TE-induced expression of inflammatory cytokines (*Il1b*,* Tnf* ), regulatory cytokines (*Il10*,* Tgfb*), and chemokines (*Ccl2*,* Ccl5*,* Cxcl10*,* Cxcl13*) were not significantly different between *Irf7*^−/−^ and *Irf7*^fl/fl^*Lyz2*-cre mice aside from *Cxcl10* at 7dpi (Fig. 4), indicating that the nature of inflammation in the CNS between *Irf7*^−/−^ and *Irf7*^fl/fl^*Lyz2*-cre mice is largely similar and further suggests that myeloid-specific deletion of *Irf7* recapitulates the immune response seen in *Irf7*^−/−^ mice.

**Fig. 4:**
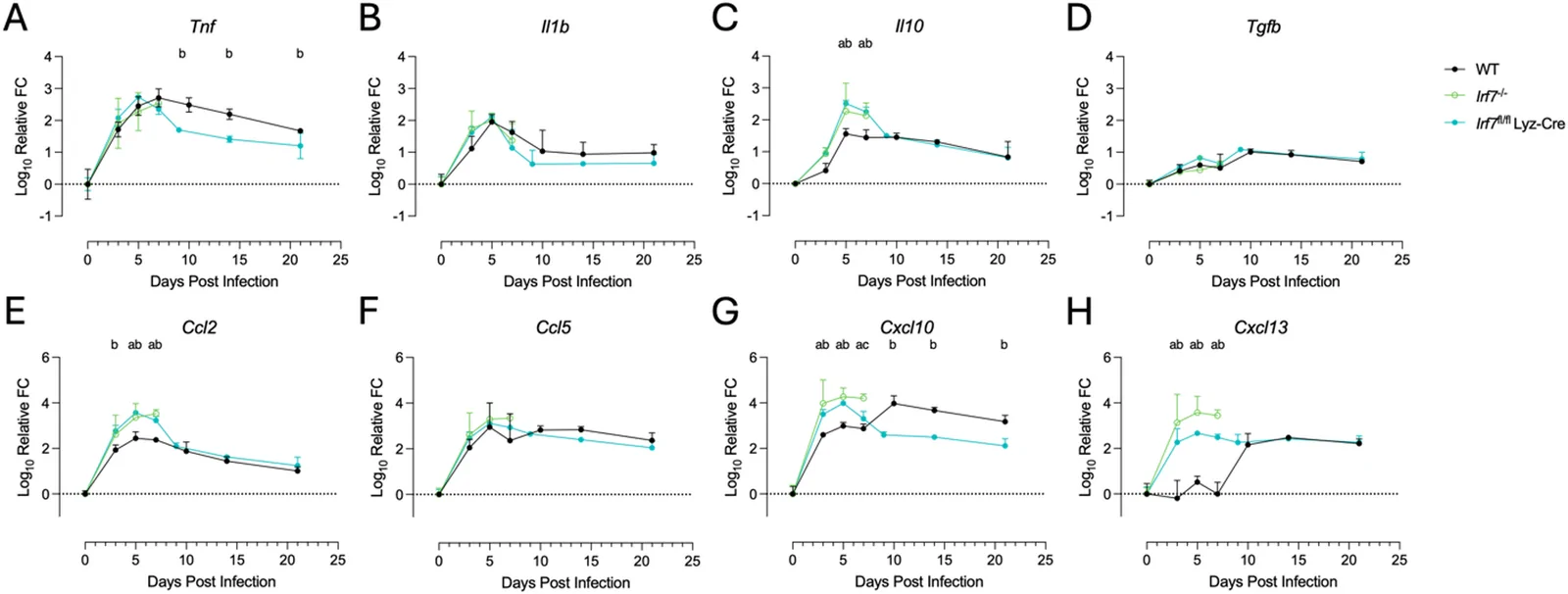
Cytokine and chemokine expression in the brains of WT, Irf7^−/−^, and Irf7^fl/fl^ Lyz2-cre mice. Cytokine (**A**-**D**) and chemokine (**E**-**H**) expression in the brain was measured by qRT-PCR. Expression was normalized to Gapdh and compared to uninfected controls of each genotype. Data are presented as the mean ± SD for three individual mice per time point per genotype. Data labeled with letters differ significantly at that time point (*P* < 0.05). No indicator: non-significant, a: WT vs. Irf7^−/−^, b: WT vs. Irf7^fl/fl^ Lyz2-cre, c: Irf7^−/−^ vs. Irf7^fl/fl^ Lyz2-cre, d: three-way difference; Tukey’s (0–7 dpi) or Bonferroni’s (9–21 dpi) multiple comparisons test

### Myeloid-specific *Irf7* deletion is associated with an increased CNS infiltration of inflammatory monocytes

Given the reports of enhanced inflammatory monocyte and macrophage levels in the CNS of *Irf7*^−/−^ mice [11, 29], we investigated the cellular dynamics in the brains of *Irf7*^fl/fl^*Lyz2*-cre mice.

(Fig. 5, Figure S3-S6). Cells were profiled at 5 dpi, which corresponded to the onset of paralysis in *Irf7*^−/−^ mice. Analysis gated on CD45^hi^ cells, which ensures examination of infiltrating immune cells as opposed to microglia, which are CD45^lo^ [39]. We focused on infiltrating immune cells because previous studies showed no differences in the amount or activation of microglia in the brains of WT and *Irf7*^−/−^ mice following SINV TE infection [29].

**Fig. 5:**
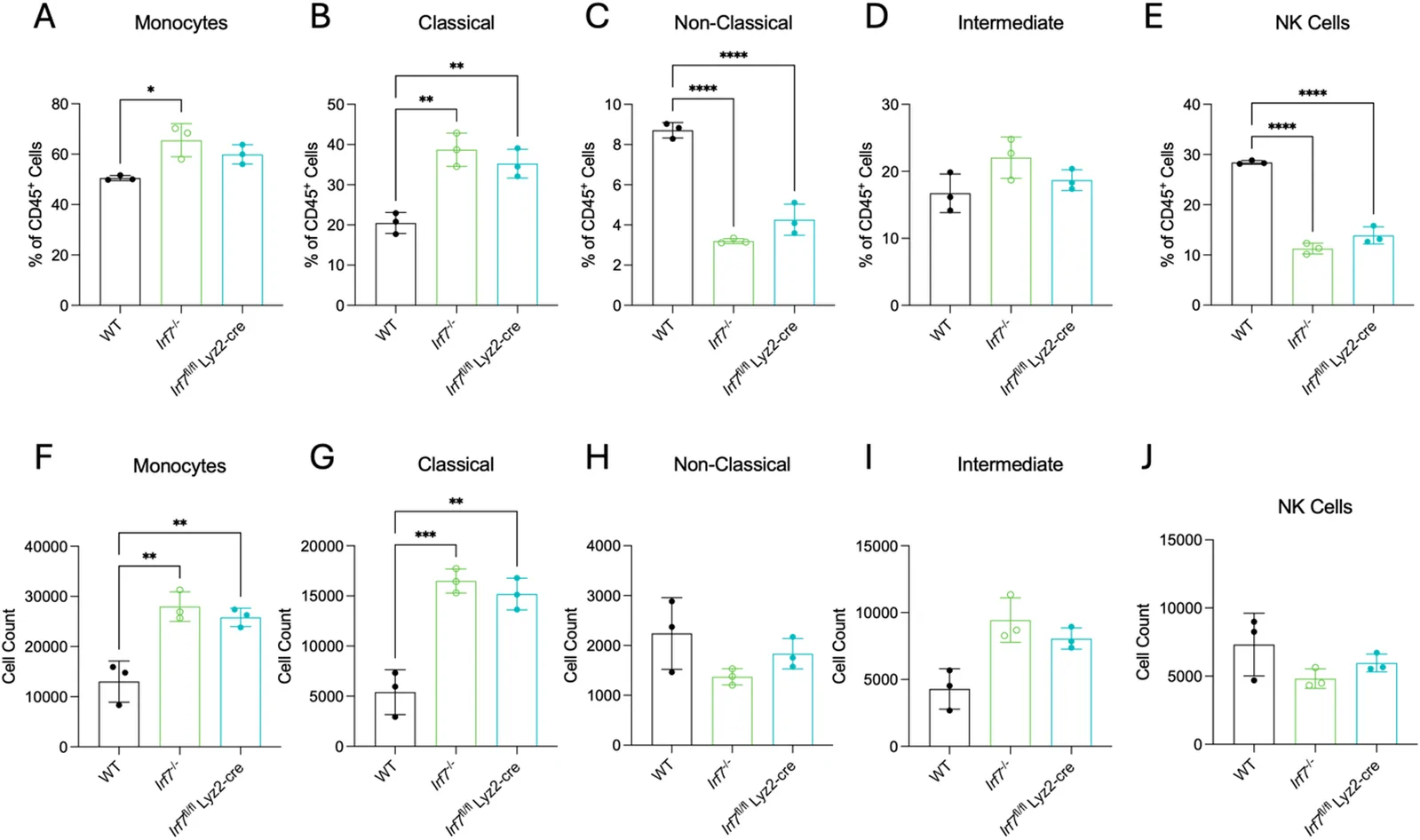
Monocytic cell infiltrate into the brain at 5dpi. Flow cytometry analysis of infiltrating monocytes isolated from the brains of mice 5 days after infection with SINV. Percentages of live CD45+ cells that were monocytes (**A**), classical monocytes (**B**), non-classical monocytes (**C**), or intermediate monocytes (**D**) with corresponding cell counts (I-L). Cells were pooled from five-seven mice per genotype for each data point from three independent experiments and all samples were stained and flowed on the same day. The data are presented as mean ± SD. No indicator, non-significant, *P < 0.05, **P < 0.01, ***P < 0.001, ****P < 0.0001; Tukey’s multiple comparisons test

The innate compartment showed the largest differences between WT and *Irf7*^−/−^ mice (Fig. 5, Figure S4). Proportions of total monocytes were higher in *Irf7*^−/−^ mice compared to WT, and *Irf7*^fl/fl^Lyz2-cre mice had an intermediate amount that did not reach statistical significance when compared to either WT or *Irf7*^−/−^ mice (Fig. 5A). Examination of monocyte subsets (Fig. 5B and D) revealed increased proportions of classical and decreased proportions of non-classical monocytes in *Irf7*^−/−^ mice compared to WT. These results are recapitulated in *Irf7*^fl/fl^*Lyz2*-cre mice, suggesting that myeloid-specific deletion of *Irf7* is sufficient to alter monocytic polarization. There were no differences in intermediate monocyte proportions between groups. Natural killer (NK) cell proportions showed a stark difference in *Irf7*^−/−^ and *Irf7*^fl/fl^*Lyz2*-cre mice and were reduced by roughly half compared to WT mice (Fig. 5E). Corresponding cell counts largely reflect the same trends seen with proportional differences (Fig. 5F-J). These data support the notion that IRF7 regulates the inflammatory signature of myeloid cells responding to SINV TE infection, and dysregulated inflammation in the absence of IRF7 likely results in more severe disease and death.

Slight alterations in the DC compartment were also detected (Figure S4). Total proportions of DCs were decreased in *Irf7*^fl/fl^ *Lyz2*-cre mice compared to WT and *Irf7*^−/−^ mice (Figure S4A), which had similar amounts of DC infiltrate. When stratified by DC subsets, although cDC1 is a very small population in the brain at this time point, proportions were lower in both *Irf7*^−/−^ and *Irf7*^fl/fl^*Lyz2*-cre mice compared to WT (Figure S4B). cDC2 was found in higher proportions in *Irf7*^−/−^ mice compared to *Irf7*^fl/fl^*Lyz2*-cre, but neither differed from WT proportions (Figure S4C). Interestingly, despite striking differences in IFNα levels (Fig. 3F), pDCs – important producers of IFNα – trended higher at 5 dpi but were not significantly elevated in the brains of *Irf7*^fl/fl^*Lyz2*-cre animals (Figure S4D).

In the lymphocyte compartment, no differences were observed between genotypes (Figure S5, Figure S6), likely because 5 dpi is a time point that precedes a fully developed adaptive immune response. Taken together, at 5dpi, the results strongly indicate that IRF7-deficiency has the largest effect on the innate immune cell compartment, namely monocytes and NK cells, and these alterations likely contribute to disease pathogenesis. Further, *Irf7*^fl/fl^*Lyz2*-cre mice phenocopying full-body *Irf7*^−/−^ mice despite robust expression of IFNα suggests that there is a noncanonical role for IRF7 in cell polarization and recruitment into the CNS.

### Loss of IRF7 expression in myeloid cells does not affect downstream IFN signaling or induction of known clearance factors

Lastly, to confirm that severe disease in *Irf7*^fl/fl^*Lyz2*-cre mice was driven by the loss of IRF7 as opposed to altered downstream signaling, we assessed ISG expression by qRT-PCR (Fig. 6A and C) and found no differences in the expression of *Isg15* or *Ifit1* between WT, *Irf7*^−/−^, and *Irf7*^fl/fl^*Lyz2*-cre mice, and expression of *Rsad2* was slightly higher in both *Irf7*^−/−^ and *Irf7*^fl/fl^Lyz2-cre mice compared to WT mice at some time points. These findings indicate downstream ISG induction is likely unaffected in the *Irf7*^fl/fl^Lyz2-cre animals, and that elevated ISG expression is likely a result of elevated type I IFN production in *Irf7*^−/−^ and *Irf7*^fl/fl^*Lyz2*-cre mice (Fig. 3F and G).

**Fig. 6:**
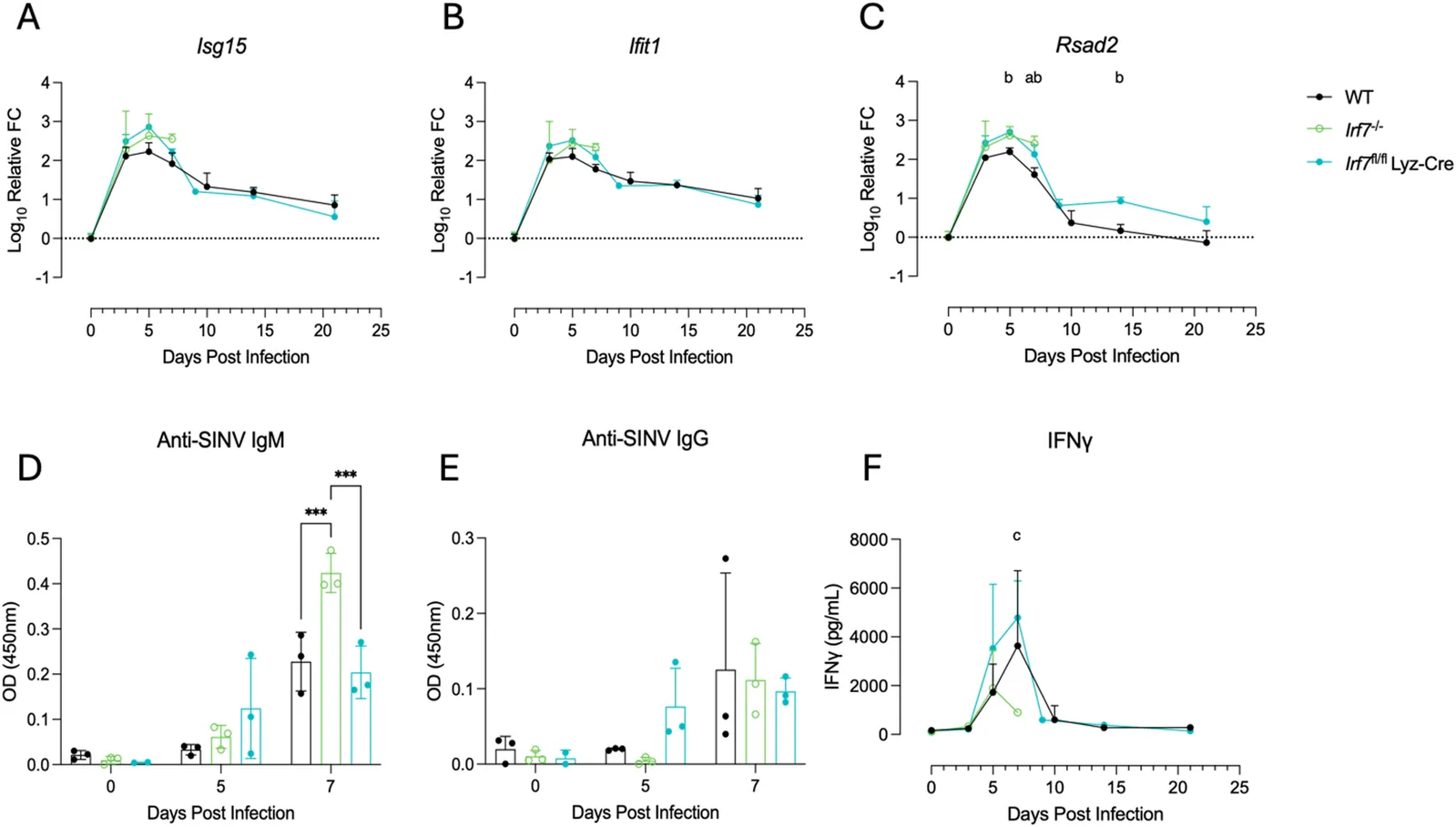
ISG, IFNγ, and virus-specific antibody levels in the brains of WT, Irf7^−/−^, and Irf7^fl/fl^ Lyz2-cre mice. ISG expression in the brain was measured by qRT-PCR (**A**-**C**). Expression was normalized to Gapdh and compared to uninfected controls of each genotype. Data are presented as the mean ± SD for three individual mice per time point per genotype. IFNγ (**D**) and virus-specific IgM (**E**) and IgG (**F**) levels were measured by ELISA. Data are presented as the mean ± SD from three-four individual mice per time point per genotype. Data labeled with letters differ significantly at that time point (*P* < 0.05). No indicator: non-significant, a: WT vs. Irf7^−/−^, b: WT vs. Irf7^fl/fl^ Lyz2-cre, c: Irf7^−/−^ vs. Irf7^fl/fl^Lyz2-cre, d: three-way difference; Tukey’s (0-7dpi) or Bonferroni’s (9-21dpi) multiple comparisons test. No indicator, non-significant, *P < 0.05, **P < 0.01, ***P < 0.001, ****P < 0.0001 (black, WT vs. Irf7^−/−^; blue, WT vs. Irf7^fl/fl^ Lyz2-cre; green, Irf7^−/−^ vs. Irf7^fl/fl^ Lyz2-cre); Tukey’s (0–7 dpi) or Bonferroni’s (9–21 dpi) multiple comparisons test

IFNγ and virus-specific antibody are crucial mediators of SINV clearance from the CNS [36–38, 40–42]. To assess how IRF7-deficiency in myeloid cells affects their expression, we measured IFNγ and SINV-specific antibody levels in the brains of WT, *Irf7*^−/−^, and *Irf7*^fl/fl^*Lyz2*-cre mice (Fig. 6D and F). IFNγ levels were identical among groups over the course of infection except for lower amounts in *Irf7*^−/−^ mice at 7 dpi (Fig. 6D). Virus specific IgM (Fig. 6E) and IgG (Fig. 6F) titers in the brains were also similar across conditions aside from, again, *Irf7*^−/−^ at 7dpi, which had significantly higher amounts of IgM. Because these mice do not differ from WT with regard to production of factors known to be required for SINV clearance, it further supports the notion that mortality is a consequence of the lack of IRF7 itself as opposed to the inability to express other products downstream of IFNα signaling.

## Discussion

The importance of IRF7 for survival from alphavirus encephalomyelitis is underscored by the fact that *Irf7*^−/−^ mice develop severe paralysis and death from infection while WT mice have mild disease and recover [11]. While *Irf7*^−/−^ mice are commonly employed as a surrogate for a pan-IFNα subtype knockout [15], the mechanistic basis for phenotypes observed in *Irf7*^−/−^ models remains unclear as it can be attributed to the loss of IRF7, IFNα, or both. Based on the data presented here, we postulate that IFNα is neuroprotective through some mechanism not directly examined here, and this explains why some mice are able to survive infection when supplemented with IFNα (Fig. 2), but the incomplete protection suggests that this is a phenomenon independent from IRF7 deficiency. Therefore, while the loss of either IFNα or IRF7 contributed to altered disease outcome in *Irf7*^−/−^ mice, the ability to produce IRF7 is ultimately a stronger determinant of outcome than IFNα production. It is also possible that a specific subtype or combination of subtypes of IFNα beyond what was included in the injections is required for survival, resulting in the incomplete protection observed with IFNα supplementation (Fig. 2).

To characterize the contribution of IRF7 in myeloid cell trafficking and polarization, we used a Cre-Lox system and conditionally deleted *Irf7* in myeloid cells using *Irf7*^fl/fl^*Lyz2*-cre mice. These mice had high levels of death from SINV infection that mirrored what was observed in *Irf7*^−/−^ mice (Fig. 3), underscoring the importance of IRF7 expression in myeloid cells on disease outcome. It should be noted that Lyz2-cre mice do not express Cre recombinase to high levels in microglia, likely due to their yolk sac origin [43, 44]. Additionally, although these mice have Cre activity in neurons [45], the general survival of *Irf7*^fl/fl^*Syn1*-cre indicates that any effects from neuronal IRF7 deficiency is not the main cause of death. Similarly, astrocytic IRF7 deficiency using *Irf7*^fl/fl^*Gfap*-cre mice do not result in excess mortality. Thus, we speculate that the phenotype seen in *Irf7*^fl/fl^*Lyz2*-cre mice is attributed to IRF7 expression in circulating myeloid cells that are recruited to the CNS during SINV infection as opposed to effects from CNS-resident microglia.

The disease manifestations in *Irf7*^fl/fl^*Lyz2*-cre mice are strikingly similar to that noted for *Irf7*^−/−^ animals (Fig. 3). Most importantly, severe disease developed in the *Irf7*^fl/fl^*Lyz2*-cre mice despite robust expression of IFNα (Fig. 3F), dispelling our initial hypothesis that IFNα is required for survival from SINV TE infection. These results instead suggest that paralysis and death is possibly mediated by infiltrating IRF7-deficient monocytes from the periphery. To ensure that other aspects of the immune response mirror that of *Irf7*^−/−^ mice, we examined ISG and inflammatory cytokine/chemokine expression in the brain, which were comparable in *Irf7*^−/−^ and *Irf7*^fl/fl^Lyz2-cre mice (Fig. 4), underscoring how *Irf7*^fl/fl^Lyz2-cre mice phenocopy *Irf7*^−/−^ mice with regard to the inflammatory response. *Cxcl10* transcripts were higher in *Irf7*^−/−^ mice compared to WT and *Irf7*^fl/fl^ *Lyz2*-cre mice, which could account for the drop off in mortality of *Irf7*^−/−^ mice at that time point. Of note: gene expression differences may not account for protein-level changes and is a limitation of these data; therefore, it is possible that protein levels of ISGs and cytokines may be different from the transcripts. We also checked levels of IFNγ and virus-specific antibody in the CNS (Fig. 6), both factors important for clearance of SINV infection [34–37, 40–42, 46], and levels were not significantly different from those of WT mice. This supports the hypothesis that the disease manifestation is driven by the actions of IRF7-deficient myeloid cells as opposed to dysregulation of other aspects of the immune response important for resolution of infection.

Both the *Irf7*^−/−^ and *Irf7*^fl/fl^*Lyz2*-cre showed notable increases in the levels of CNS-infiltrating monocytes (Fig. 5). There are increased proportions of monocytes in both *Irf7*^−/−^ and *Irf7*^fl/fl^*Lyz2*-cre mice, driven by an increase of classical monocytes and reduction of non-classical monocytes (Fig. 5). Broadly speaking, classical monocytes are inflammatory and non-classical monocytes are anti-inflammatory [47, 48]. Therefore, these results suggest that IRF7 deficiency skews monocytes toward an inflammatory signature, and this is consistent with other studies showing the role of IRF7 in suppressing inflammatory gene expression in myeloid cells [24–28]. Additionally, in a model of traumatic brain injury, depletion of peripheral monocytes is neuroprotective, underscoring the pathologic effects of monocyte recruitment during neuroinflammation [49]. Taken together, it seems probable that disease pathogenesis in *Irf7*^−/−^ mice is driven by an IRF7 deficiency in myeloid cells, resulting in their increased inflammatory signature that causes neuronal damage. Additional work to validate and characterize the mechanism by which this occurs is the focus of future studies.

Lastly, it is possible that heightened IFNβ and/or IFNα expression in *Irf7*^−/−^ and *Irf7*^fl/fl^ *Lyz2*-cre mice (Fig. 3F and G) is the mediator of increased inflammation in the CNS following SINV TE infection. IFNα especially is associated with inflammation-driven neurotoxicity due to high or prolonged exposure [50]; therefore, it is possible that increased type I IFN production in *Irf7*^−/−^ and *Irf7*^fl/fl^*Lyz2*-cre mice underlies heightened inflammation and disease severity in the absence of IRF7. Direct assessment of this by experimental depletion of IFN in the brain is difficult due to the impermeability of the BBB to antibodies that would deplete IFN. However, preliminary studies examining *Irf7*^−/−^ *Ifnb*^−/−^ double knock-out (DKO) mice showed that IFNβ deficiency in *Irf7*^−/−^ mice led to accelerated disease progression without a reduction in severity after SINV TE infection (**Figure S7**), suggesting that heightened IFNβ expression in *Irf7*^−/−^ mice is not associated with disease severity and is instead beneficial for delaying disease onset, but further investigation is necessary to definitively rule out the effects of heightened IFNα and IFNβ. Additionally, while heightened IFNα is neurotoxic in other contexts, peak IFNα expression does not co-occur with peak disease in *Irf7*^fl/fl^*Lyz2*-cre mice following SINV TE infection (Fig. 3), and increased classical monocyte infiltration in the absence of IRF7 production occurs whether IFNα is present or not (Fig. 5). Therefore, it is likely that heightened inflammation in *Irf7*^−/−^ and *Irf7*^fl/fl^ *Lyz2*-cre mice is associated with altered innate cell infiltration, and higher-than-WT expression of IFNα in *Irf7*^fl/fl^ *Lyz2*-cre mice is a byproduct of increased viral replication in the CNS (Fig. 3). We speculate that IFNα in *Irf7*^fl/fl^ *Lyz2*-cre mice is produced by microglia, as *Lyz2*-cre mice do not express Cre in those cells [43, 44].

In summary, these results show that the phenotype observed in *Irf7*^−/−^ mice following SINV TE infection of the CNS is driven by two factors, and we propose the following timeline of events: The effects of defective IFNα expression are seen first, resulting in unchecked virus replication and extensive spread throughout the CNS. This widespread infection inevitably causes a stronger inflammatory immune response. As IFN expression wanes, the phenotype then becomes driven by IRF7-deficiency. In the absence of IRF7, the cells that are recruited to the CNS after uncontrolled virus replication are biased toward an inflammatory phenotype. This results in heightened immune-mediated damage to neuronal cells, eventually causing the death of the host. These observations provide a more detailed timeline of recovery from SINV infection and highlight the importance of time after infection for selecting treatment options.

## Supplementary Information

Supplementary Material 1.

## Data Availability

The RNAseq data for this study are available in SRA under BioProject number PRJNA1472213. The analysis packages used were all publicly sourced. Processed counts, code, and metadata are available on GitHub at https://github.com/b-hnguyen/sinv-brain-rnaseq.
