# Supplementary material for: IRF7 deficiency in myeloid cells results in increased disease severity and death during alphavirus encephalomyelitis despite intact expression of type I interferons
Source: J Neuroinflammation. 2026 Jun 25;23:328. doi: 10.1186/s12974-026-03936-7 (PMC13625333; doi:10.1186/s12974-026-03936-7)
Supplement: Supplementary file 1 [file 12974_2026_3936_MOESM1_ESM.pdf]

Table S1

Cell Type	Markers
T cells	$CD3^+$
CD8 T cells	$CD3^+$ $CD4^-$ $CD8^+$
CD4 T cells	$CD3^+$ $CD4^+$ $CD8^-$
NK Cells	$CD3^-$ $NK1.1^+$
B cells	$CD19^+$
B2 cells	$CD19^+$ $CD23^+$
B1 cells	$CD19^+$ $CD23^-$ IgM^+ $CD43^+$
B1a cells	$CD19^+$ $CD23^-$ IgM^+ $CD43^+$ $CD5^+$
B1b cells	$CD19^+$ $CD23^-$ IgM^+ $CD43^+$ $CD5^-$
Dendritic cells	$CD11c^+$ $MHC\text{-}II^+$
Common DC 1	$CD11c^+$ $MHC\text{-}II^+$ $CD11b^-$ $CD103^+$
Common DC 2	$CD11c^+$ $MHC\text{-}II^+$ $CD11b^+$ $CD103^-$
Plasmacytoid DC	$CD11c^+$ $MHC\text{-}II^-$ $SiglecH^+$
Monocytes	$CD11b^+$ $F4/80^-$
Classical monocytes	$CD11b^+$ $F4/80^-$ $Ly6C^+$ $CD43^-$
Non-classical monocytes	$CD11b^+$ $F4/80^-$ $Ly6C^-$ $CD43^+$
Intermediate monocytes	$CD11b^+$ $F4/80^-$ $Ly6C^+$ $CD43^+$

Table S1 – Cell population definitions for flow cytometry
Defining markers used to identify cell types profiled by flow cytometry.

Figure S1

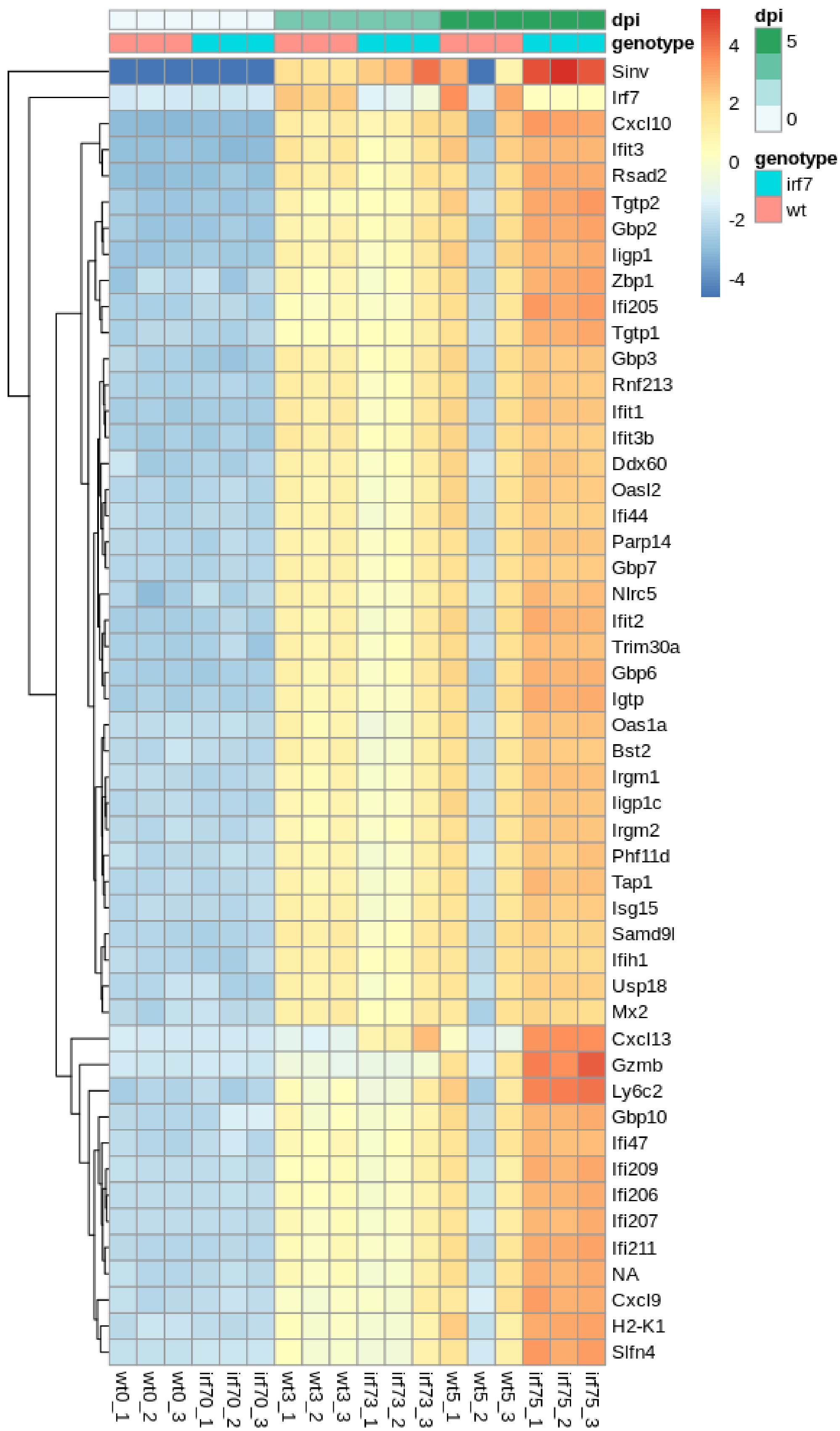

Figure S1 – Quality control of RNA sequencing data

Top 50 DEGs between WT and *Irf7*$^{-/-}$ mice, striated by individual samples and grouped by genotype and infection status. Fold change is represented as a gradient from blue (down-regulation) to red (up-regulation). Sample wt5_2 was excluded from the final analyses due to the lack of SINV reads.

Figure S2

A

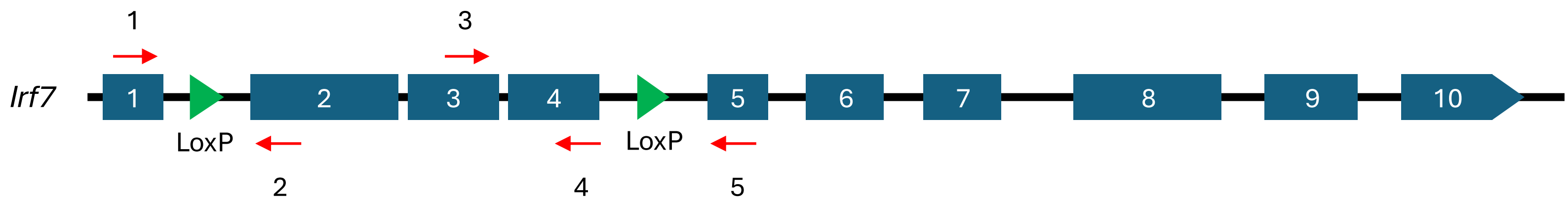

B

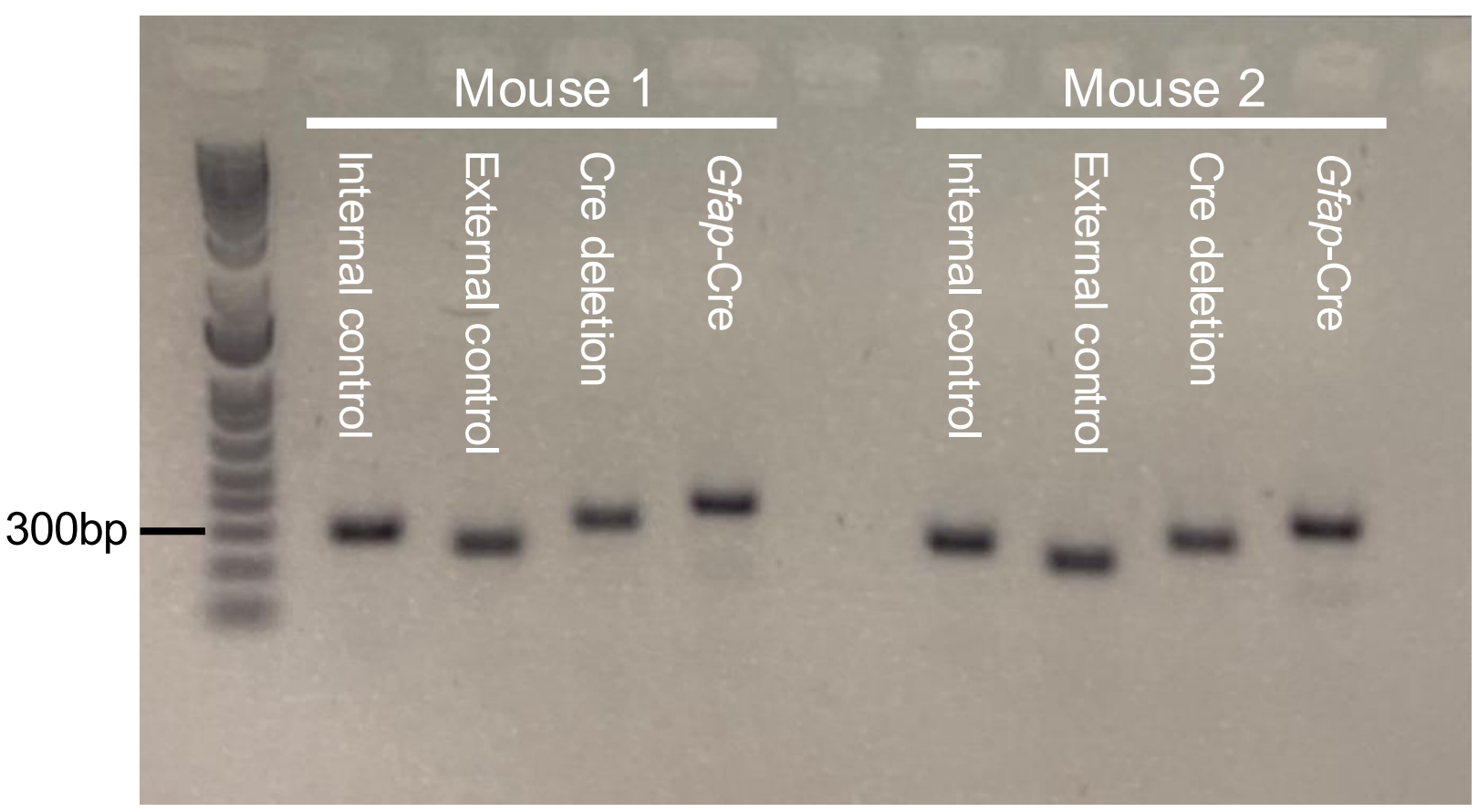

C

Band	Primers	Size
Internal control	3 & 4	301bp
External control	1 & 2	262bp
Cre deletion	1 & 5	304bp
Gfap-cre	N/A	400bp

D

ssDNA Sequence

GGGGAGGGGAGATTTGGTTACGATGCCCAAGCAGTTCCAAAAGTTCTCTATTTCTCATACCCTCTGTTCCCAAACCACCAGGT
ACCTCACTCAGCCCAAGGCTCTGACTTATAACTTCGTATAATGTATGCTATACGAAGTTATAGCAGGAAGTGTGCACTCCCCCT
CCACCCCGACACACACACACTTTGGAAGGAGAGGAACTGAATCTACAACTAGTTTCAAAAACCACAAATCAAACCATTGAAAC
CACCAAAATGACAGAGGCCATGTACCTCCCTGGCCCATCCCTGTTTGTGGCACTGCTCACCAGTAGATCCAAGCTCCCGGC
TAAGTTCGTACACCTTATGCGGATCAACTGGATCCCCTGAATTGTCTTGGCGCAAGATAAAACGCCCTGTGCTGTGGAGTGCA
CAGCGGAAGTTGGTCTTCCAGCCTCTTCGCTCTCTTCGCTCAGCAGCCTCAGCCTCTGGGGGTGGCAGGTTAACTCCACTAG
GTGGCCACCTCCCTCGGGCCACAGCCCAGGCCTGAAGGAGAGGAGAGAGCATGGGTTGAGGCTGGATTCCCCCAGGAGT
TCTGGCAGATTATAAAGAGGGCAGGACTGGGTACCACCTTGAAGATCTGTGCATCTTCTTCATCCAGATCCCTACGACCGAAA
TGCTTCCAGGGTACGCGGAAGACTGTGCGAGCCTCGTTCAGCCACTGCAGCCCCTCGTACTGGCCGCTGCTGACCTCCCC
CAATAGCCAGTCTCCAAACAGCACTCGCTGGACCCCCCTGCAGGCAGAGCAGCCACGCTGGGGTTAGGGCTAATGTAAGG
GACACAGGACCCAATACAACCAGGGTAGACATGTTCTCACCTCACTTCAGCCATTGCTCCGTCTACACAGGCAGTCTGGGAG
AATCAAAGTAGATGCTACTACTCTGTGATGCCTTGTAGTCACTGGAGCAGTCAGCTATTGCAAATAAGGCCCCTTGGGTGCCTC
TATGGTTTTAGGTTGTAGCCACAGATAGTGTGACCAAGTTTCACAAATGTGTTTTAAACGTTACAAGTGTTGGTAACAGGTAGGAA
TTTCAGTTTCACAAGTGTGACCCAGGTATTAGGGTGTTGAGGGTCACAGATGAGGTTTGAGAACTTGTGGTCACCTAGGTTCAG
AACTCAGTGTAGATTCCTCTGCCAAGGTGGCTGTAGATGTGACTACAGATGTAGCGGGAGCGCACACGTGATGTCACAGGTG
TTAATCCAGCGTCCGAGCCTGGATGGGCTGTCACAGGCTGGACCCTGCGAAGACACAAAAGGGGCGGGCCCAGACGAGTA
AAACGAAACTGCATCTCAGGAAAATGAAACCTAAACAGTCTAAACAGCGCCCGGTAGCTGCTGGCTTCCCGCTGTCTCCACT
GTCTCCATGCCTACCAGTGCAGTCCTCAAATAACTTCGTATAATGTATGCTATACGAAGTTATACTACACCCGACCCTTACTCAG
ATCCCAATCCCTGCAGACCCAATTGCAAATCCTGCCGGGACGTCTGCCCTCCTGTTTCCCCCGAGCTTCTCTTCAC TGCCC

Figure S2 – Generation of *Irf7*$^{fl/fl}$ mice

The *Easi*-CRISPR protocol was used to generate *Irf7*$^{fl/fl}$ mice (31, 32). (A) Schematic depicting where LoxP (green arrows) sites were inserted in the introns (black lines) between exons (blue boxes) 1 and 2, and 4 and 5, of the *Irf7* locus with primers for validation of Cre-mediated recombination shown (red arrows). LoxP sites were inserted within sequences used for targeting gRNAs to ensure that the donor strand was not cut by Cas9 following homology directed repair. (B) PCR gel validating Cre-mediated recombination in the brains of two different *Irf7*$^{fl/fl}$ mice crossed to Gfap-cre mice. (C) Primer combinations and expected amplicon lengths in basepairs (bp).

Figure S3

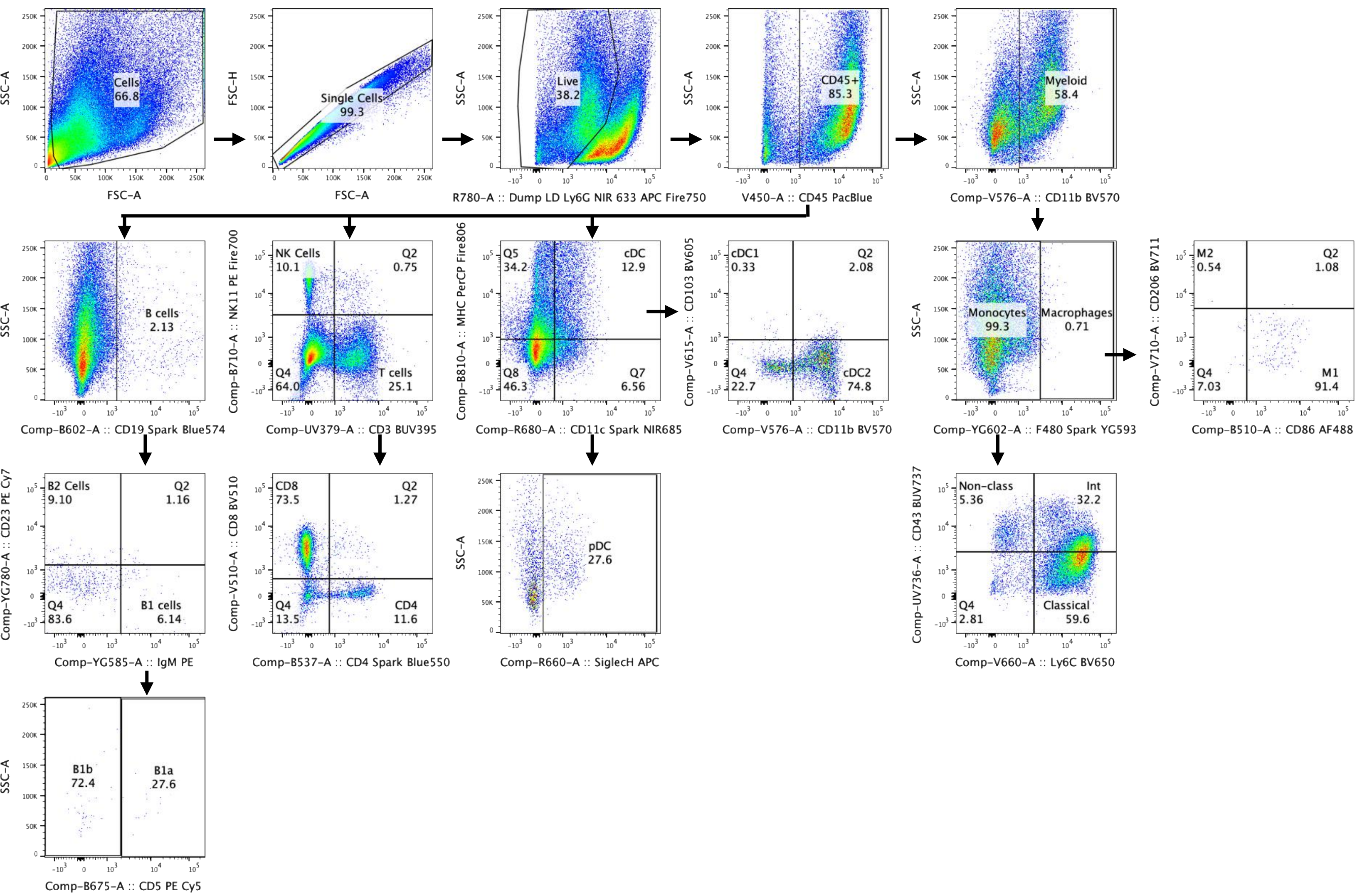

Figure S3 – Flow cytometry gating
Flow cytometry gating strategy for all cell populations.

Figure S4

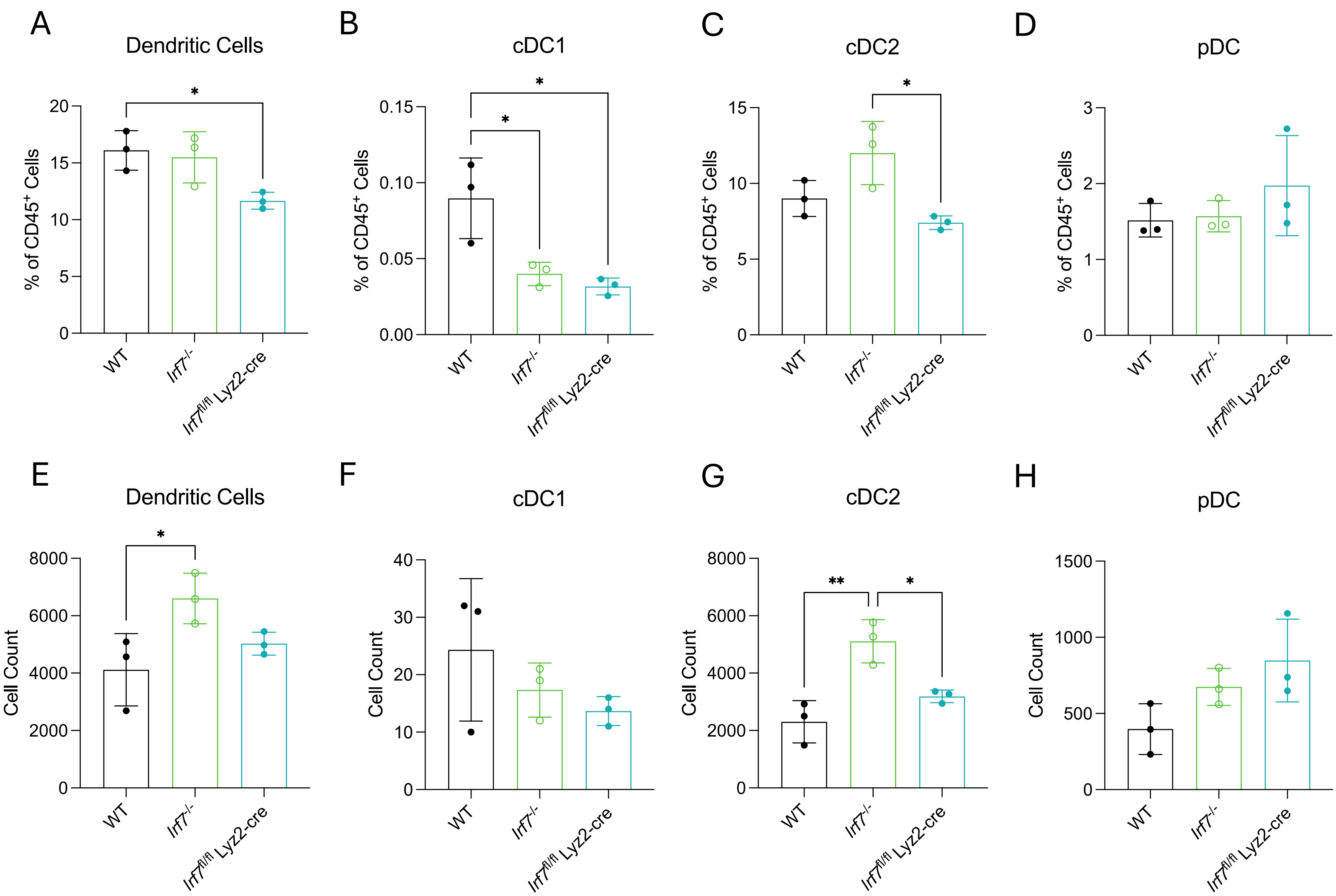

Figure S4 – Dendritic and NK cell infiltrate into the brain at 5 dpi
Flow cytometry analysis of infiltrating dendritic cells (DC) isolated from the brains of mice 5 days after infection with SINV. Percentages of CD45$^+$ cells that were DCs (A), classical DC 1 (B), classical DC 2 (C), or plasmacytoid DCs (D) with corresponding cell counts (E-H). Cells were pooled from five-seven mice per genotype for each data point, and the data are presented as mean ± SD. No indicator, non-significant, *$P < 0.05$, **$P < 0.01$, ***$P < 0.001$, ****$P < 0.0001$; Tukey's multiple comparisons test.

Figure S5

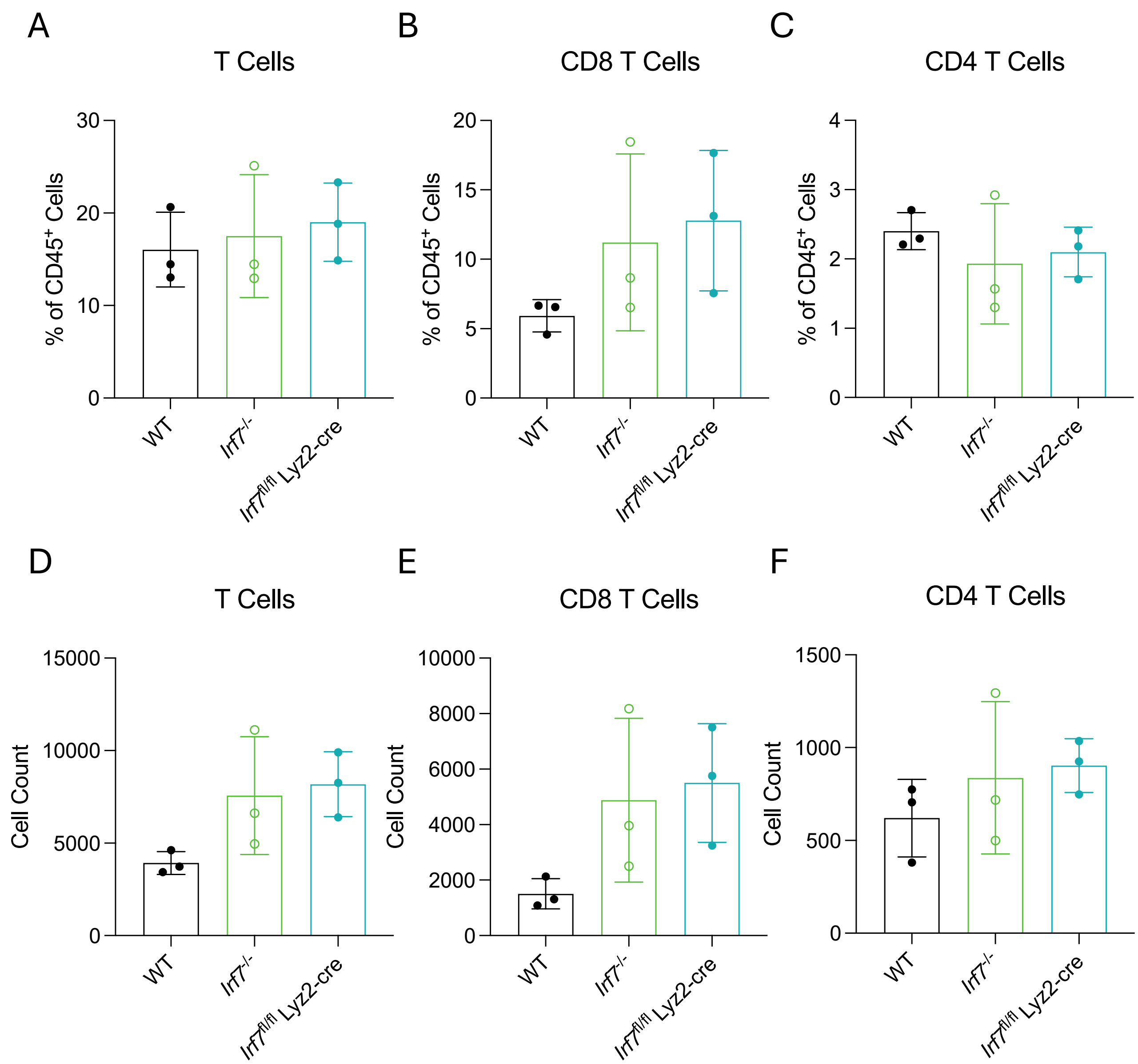

Figure S5 – T cell infiltrate into the brain at 5 dpi
Flow cytometry analysis of infiltrating T cells isolated from the brains of mice 5 days after infection with SINV. Percentages of $CD45^+$ cells that were T cells (A), CD8 T cells (B), or CD4 T cells (C) with corresponding cell counts (D-F). Cells were pooled from five-seven mice per genotype for each data point, and the data are presented as mean ± SD. No indicator, non-significant, *$P < 0.05$, **$P < 0.01$, ***$P < 0.001$, ****$P < 0.0001$; Tukey's multiple comparisons test.

Figure S6

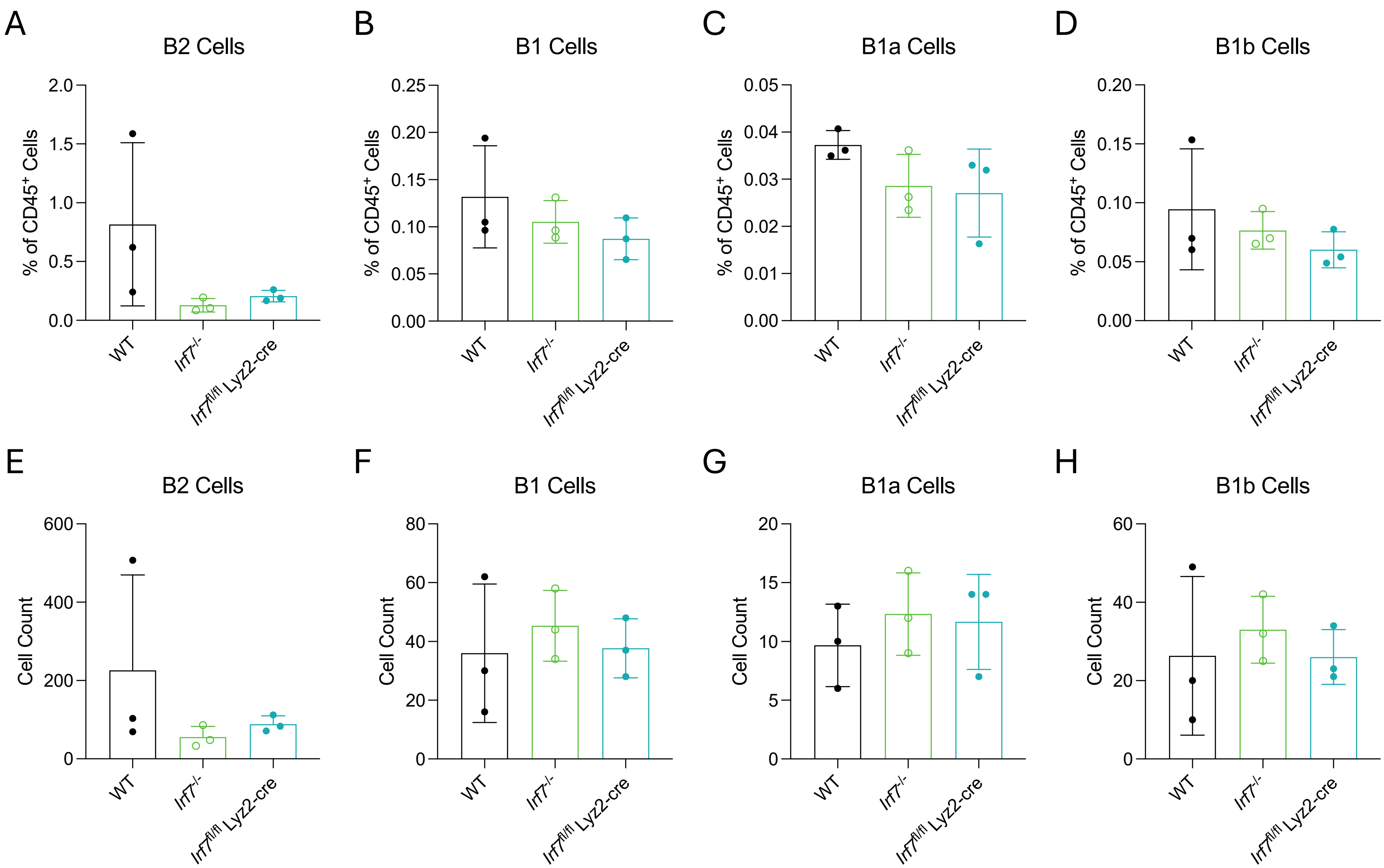

Figure S6 – B cell infiltrate into the brain at 5dpi
Flow cytometry analysis of infiltrating B2 cells (A, E) and B1 cells (B-D, F-H) isolated from the brains of mice 5 days after infection with SINV. Percentages of $CD45^{+}$ cells that were B2 cells (A), B1 cells (B), B1a cells (C), or B1b T cells (D) with corresponding cell counts (E-H). Cells were pooled from five-seven mice per genotype for each data point, and the data are presented as mean ± SD. No indicator, non-significant, *$P < 0.05$, **$P < 0.01$, ***$P < 0.001$, ****$P < 0.0001$; Tukey's multiple comparisons test.

Figure S7

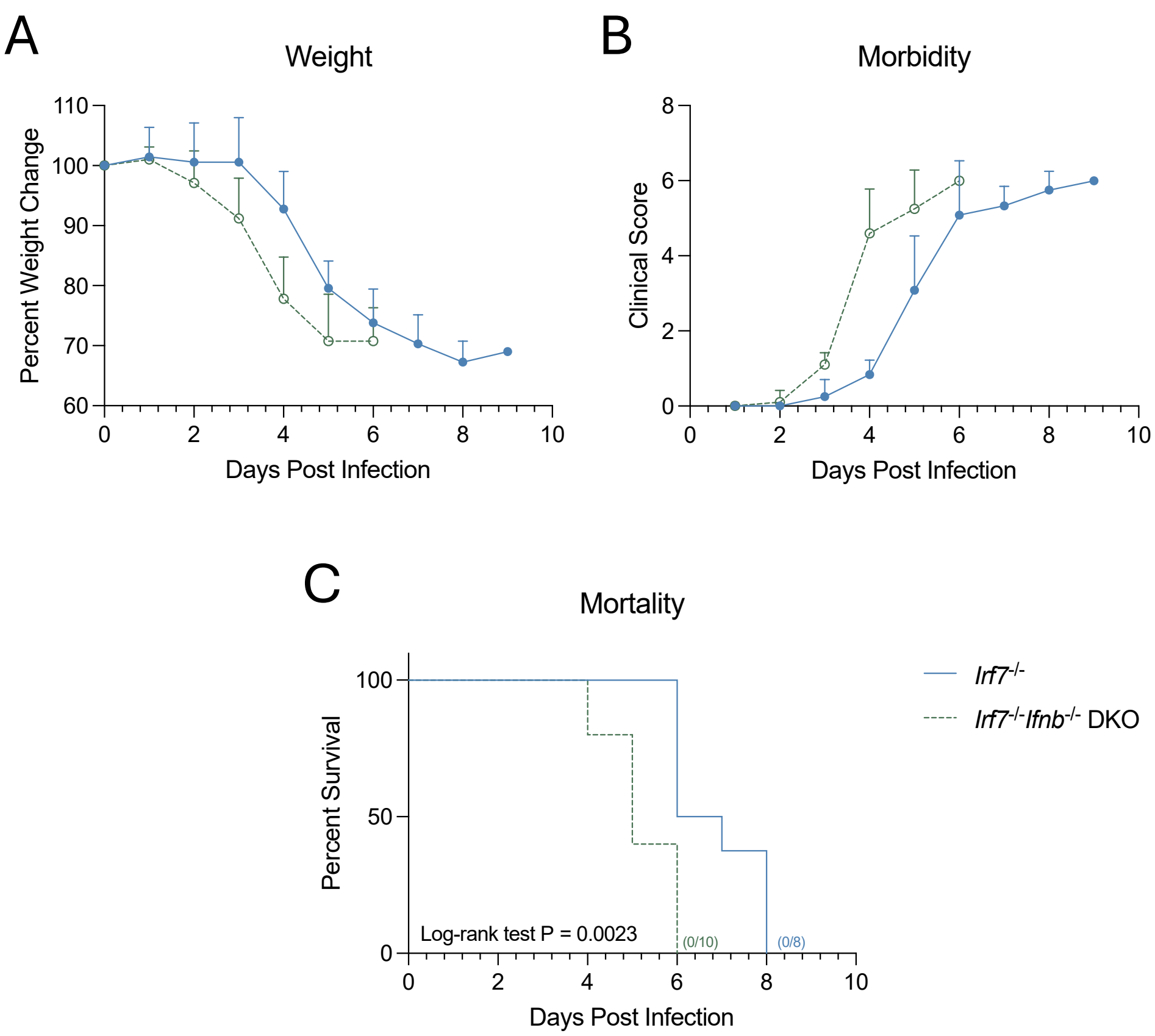

Figure S7 – Disease manifestation in *Irf7$^{-/-}$ Ifnb$^{-/-}$* mice
5-8-week-old male and female *Irf7$^{-/-}$* or *Irf7$^{-/-}$ Ifnb$^{-/-}$* mice were intracerebrally inoculated with 10^3 PFU of SINV TE. Mice were monitored daily for signs of weight loss (A), clinical disease (B), and death (C). The clinical scoring system was: 0 = clinically normal; 1 = abnormal gait and/or ataxia; 2 = mild hunched posture with occasional two-leg stance but normal demeaner and ambulation; 3 = markedly hunched posture with minimal ambulation or obtunded; 4 = unilateral hindlimb paralysis; 5 = bilateral hindlimb paralysis or moribund; 6 = death. Survival was assessed using a Kaplan–Meier analysis and the log-rank test.
